# Laticifers in Sapindaceae: Structure, Evolution and Phylogenetic Importance

**DOI:** 10.3389/fpls.2020.612985

**Published:** 2021-01-18

**Authors:** Maria Camila Medina, Mariane S. Sousa-Baena, Erika Prado, Pedro Acevedo-Rodríguez, Pedro Dias, Diego Demarco

**Affiliations:** ^1^Departamento de Botânica, Instituto de Biociências, Universidade de São Paulo, São Paulo, Brazil; ^2^Department of Botany, National Museum of Natural History, Smithsonian Institution, Washington, DC, United States; ^3^Escola de Artes, Ciências e Humanidades – EACH Universidade de São Paulo, São Paulo, Brazil

**Keywords:** laticifers, Paulliniodae, Sapindaceae, evolution, latex, ontogeny, callose, suberin

## Abstract

Laticifer occurrence and structure are poorly known in Sapindaceae. Occurrence is likely underestimated owing to the low production of latex in most species. We investigated 67 species from 23 genera of Sapindaceae to verify laticifer occurrence and their structural, developmental and chemical features, as well as their evolutionary history in the family. Shoots were collected from herbarium and fresh specimens for histological analyses. Three characters derived from laticifer features were coded and their ancestral states reconstructed through Bayesian stochastic mapping and maximum likelihood estimation. Only articulated non-anastomosing laticifers were found in Sapindaceae. Laticifers differentiate early during shoot development and are found in the cortex, phloem, and pith. Latex is mostly composed of lipids. Callose and suberin were detected in laticifer cell walls in some genera. Reconstruction of laticifer ancestral states showed that laticifers are present in most clades of Sapindaceae with some reversals. Callose in the laticifer cell wall was found exclusively in *Serjania* and *Paullinia* (tribe Paullinieae), a character regarded as independently derived. Occurrence of laticifers in Sapindaceae is broader than previously reported. Articulated non-anastomosing laticifers had five independent origins in Sapindaceae with some secondary losses, occurring in five out of six genera of Paullinieae and 10 other genera outside Paullinieae. Particularly, callose in the laticifer cell wall evolved independently twice in the family, and its occurrence may be interpreted as a key-innovation that promoted the diversification of *Paullinia* and *Serjania*. Our study suggests that laticifer characters may be useful in understanding the generic relationships within the family.

## Introduction

Laticifers have a wide distribution in the plant kingdom and are found in 40 families, which are phylogenetically distantly related (Prado and Demarco, 2018). They occur in almost all major groups of vascular plants from ferns (e.g., *Regnellidium*) (Gouvêa Laboriau, 1952) and gymnosperms (e.g., *Gnetum*) (Behnke and Herrmann, 1978; Tomlinson and Fisher, 2005) to various lineages of angiosperms. Laticifers evolved many times in the evolutionary history of terrestrial plants and predominate in tropical regions (Prado and Demarco, 2018). Fossil records indicate that such a secretory structure evolved in the beginning of Cenozoic era (Mahlberg et al., 1984), when abrupt global warming coincided with an increase of insect diversity and consequently a sharp increase in insect herbivory (Currano et al., 2008).

Comparative systematic studies of laticifers are scarce. Nevertheless, the presence of these structures is of great taxonomic importance as they may be diagnostic for some families, e.g., Araceae, Asteraceae, Moraceae, Nelumbonaceae, and Papaveraceae (Simpson, 2010). In addition, the chemical composition of latex varies and may have taxonomic applications and importance relevance in the interpretation of the evolutionary history of the groups in which they are found (Rudall, 1987). This is the case of Sapindales, a large and diverse order with representatives that exude a white secretion, usually interpreted as latex. Within Sapindales the presence of latex has been considered an unusual characteristic as it is only observed in a few species of Sapindaceae (APG, 2016). Sapindaceae is the largest family of the order, comprising ca. 144 genera and 1900 species distributed in four subfamilies: Xanthoceroideae, Hippocastanoideae, Dodonaeoideae, and Sapindoideae. Among them, Sapindoideae stand out as the most diverse subfamily with approximately 1340 species (Acevedo-Rodríguez, 2011; Muellner-Riehl et al., 2016). Acevedo-Rodríguez et al. (2017), based on molecular and morphological phylogenetic analyses, recognized the supertribe Paulliniodae in the Sapindoideae, which comprises four tribes (Paullinieae, Thouinieae, Bridgesieae, and Athyaneae).

There are a few reports of laticifers in the subfamily Sapindoideae, more specifically in the tribe Paullinieae (Weckerle and Rutishauser, 2005). With respect to the type, they are described as branched in *Paullinia carpopoda* (Ferraz and Gonçalves, 1985) and articulated non-anastomosing in *Paullinia micrantha*, *P. pseudota*, *P. trigonia*, *P. weinmannifolia*, and *Serjania pernambucencis* (Cunha Neto et al., 2017). There are no records in the literature for the presence of laticifers in the remaining tribes or clades belonging to subfamily Sapindoideae (Landrigan et al., 1994; Nacif et al., 2001; Zavaleta-Mancera et al., 2003; Suárez et al., 2004; Tamaio and Somner, 2010; Arévalo-Galarza et al., 2018).

Presence of latex in Hippocastanoideae has been reported for species of *Acer* and *Dipteronia*, but no histochemical tests were provided to characterize them as latex (Benedict, 1961; Amini et al., 2008). Within the Dodonaeoideae some anatomical studies have been performed (Paoli and Sarti, 2008; Bibi et al., 2014; AL-Aani et al., 2016; Onuminya and Adediran, 2018), but no laticifers have been found. Similarly, within the Xanthoceroideae, a monotypic subfamily endemic to China (Buerki et al., 2009), there are no reports of the presence of laticifers in the studies of floral anatomy of *Xanthoceras* (Zhou et al., 2012, 2019).

Besides the lack of studies describing the anatomical characteristics of laticifers, the abundant presence of secretory idioblasts in Sapindales as a whole hinders the correct identification of laticifers in Sapindaceae. Considering the co-occurrence of these two secretory structures in the family and their similar ontogeny (Milanez, 1959), these two structures can only be distinguished through histochemical analyses. As pointed out by Fahn (1979), the characterization of secretory idioblasts and laticifers sometimes resides only in the nature of their secretion.

Considering that the presence of laticifers, their type and their latex composition have taxonomic and systematic implications (Demarco et al., 2013), a comprehensive investigation of Sapindaceae was launched in order to evaluate the occurrence of laticifers at the generic level. Hence, using a broad sampling, we investigated the occurrence and ontogeny of laticifers, as well as latex composition, in representatives of supertribe Paulliniodae (tribes Paullinieae, Thouinieae, Bridgesieae, and Athyaneae). We also coded characters derived from anatomical observations of laticifers and secretory idioblasts and reconstructed their ancestral states to gain an initial understanding of the evolution of secretory structures in Sapindaceae. The results of this study also provide valuable taxonomic characters toward elucidating the evolutionary history within the family.

## Materials and Methods

### Sampling

Species were collected on the campus of the Universidade de São Paulo (Reserva Florestal and Fitotério) in São Paulo, Brazil. We also analyzed dry herbarium shoots obtained from several herbaria (Supplementary Table 1).

We sampled 67 species in 23 genera from three subfamilies:

(1) Sapindoideae: This subfamily is the most species rich, with various tribes and informally recognized groups. We analyzed the following tribes: Paullinieae, Thouinieae, Bridgesieae, Athyaneae, and the Melicoccus, Cupania, Litchi, and Blomia groups.
(2) Hippocastanoideae: This subfamily has four genera, i.e., *Acer*, *Dipteronia*, *Aesculus*, and *Billia*, all of which were analyzed in this study.
(3) Dodonaeoideae: This subfamily has 13 genera separated into two clades: Dodonaea group and Doratoxylon group; we only had access to *Dodonaea viscosa* from the Dodonaea group.

### Laticifer Detection

The occurrence of laticifers was detected using dry, FAA-fixed and fresh shoots, i.e., stem and leaves. For dry specimens, fragments of young stems were processed following the Smith and Smith (1942) protocol for the rehydration of herbarium materials. Subsequently, the fragments were dehydrated in a graded alcohol series and embedded in methacrylate (Meira and Martins, 2003). The material was transversely and longitudinally sectioned at 10 μm thickness in a rotary microtome and stained with toluidine blue (O’Brien et al., 1964). Slides were observed under bright field as well as polarized light. The photomicrographs were obtained using a Leica DMLB light microscope (Leica Microsystems, Wetzlar, Germany).

Sections were also obtained from fresh and fixed material using a sliding microtome Leica SM 2000R with an attached Microm KS 34 freezing unit (Thermo Fisher Scientific, Waltham, MA, United States) and freehand.

Considering that laticifers are often confused with other secretory cells such as idioblasts or sclereids, it was necessary to perform several histochemical tests to confirm the nature of the secretory cell. Since the predominant component of latex is lipid, the Sudan black B test was used to confirm the presence of laticifers. In complex cases, UV or blue light was also used as lipids fluoresce when subjected to such light. In the case of the idioblasts, previous tests (see “Latex composition” below) revealed they store phenolic compounds; thus, the ferric chloride test was used to distinguish them from laticifers. To discriminate both laticifers and idioblasts from sclereids or crystalliferous idioblasts, we used polarization microscopy since under this technique the secondary walls of the sclereids and the crystals stored in crystalliferous idioblasts show birefringence.

### Laticifer Quantitative Characterization

In order to investigate if there were significative differences in the size of laticifers among the different groups, we measured the diameter of the laticifers for supertribe Paulliniodae, *Melicoccus*, *Cupania*, *Litchi* groups, and *Dipteronia sinensis* (subfamily Hippocastanoideae). For this, 15 measurements were taken for each species using the ImageJ tool (Schneider et al., 2012). To establish if differences in the diameter of laticifers among groups and species were statistically significant, we used a two-way ANOVA test. This analysis was performed using R software (RStudio Team, 2015).

### Ontogeny

Three species of tribe Paullinieae were selected for the ontogenetic study of laticifers in shoots (*Paullinia seminuda*, *Serjania caracasana*, and *Urvillea ulmacea*). Vouchers of the collections made for this study were deposited in the SPF herbarium (USP).

Shoots were fixed in formalin-acetic acid-alcohol 50% (FAA) for 24 h (Johansen, 1940) or in buffered neutral formalin (Lillie, 1965) and then stored in ethanol 70%. Subsequently, shoot apices were isolated, dehydrated in an ascending butyl series (Johansen, 1940), and embedded in Paraplast (Leica Microsystems, Heidelberg, Germany). All samples were longitudinally and transversely sectioned using a rotary microtome and then stained with 1% astra blue and 1% safranin in 50% ethanol (Gerlach, 1984). Slides were mounted in Permount resin (Fisher Scientific, Pittsburgh, PA, United States) and photographed using a Leica DMLB light microscope.

### Latex Composition

Three species of tribe Paullinieae were selected to detect the main chemical compound classes that constitute the latex (*Paullinia seminuda*, *Serjania caracasana*, and *Urvillea ulmacea*). Sections were submitted to different treatments to investigate their chemical constitution as follows: (1) lipids: Sudan black B, Sudan IV (Pearse, 1985) and neutral red (Kirk, 1970), in bright field and under blue light, respectively; (2) neutral and acidic lipids: Nile blue (Cain, 1947); (3) terpenoids: Nadi reagent (David and Carde, 1964); (4) fatty acids: copper acetate and rubeanic acid (Ganter and Jollés, 1969, 1970); (5) phenolic compounds: ferric chloride (Johansen, 1940), potassium dichromate (Gabe, 1968), ferrous sulfate in formalin (Johansen, 1940) and autofluorescence under UV (Demarco, 2017); (6) tannins: vanillin – hydrochloric acid (Mace and Howell, 1974; Gardner, 1975); (7) alkaloids: Dragendorff’s reagent (Svendsend and Verpoorte, 1983) and Wagner’s reagent (Furr and Mahlberg, 1981); (8) polysaccharides: periodic acid – Schiff reaction (PAS) (Jensen, 1962); and (9) acidic mucilage and pectins: ruthenium red (Johansen, 1940; Gregory and Baas, 1989). The control for hydrophilic and lipophilic substances was carried out following Demarco (2017).

From the tests using the three species described above, we established the methodology for testing the remaining species. Sudan black B for lipids and ferric chloride for phenolics were the tests that showed the best results, even when applied to material from herbarium specimens, to identify laticifers and secretory idioblasts, respectively. Thus, for the remaining species sampled in this study we adopted these two histochemical tests for laticifer identification.

### Cell Wall Composition

Aniline blue (Smith and McCully, 1978) was used to detect callose in the laticifer cell wall of all species. In addition, the presence of suberin was analyzed using Sudan black B or autofluorescence microscopy under blue light and UV.

For all anatomical procedures, fresh and fixed materials were sectioned using a Leica SM 2000R sliding microtome with an attached freezing unit Microm KS 34 (Thermo Fisher Scientific, Waltham, MA, United States) and freehand, the slides were mounted in distilled water and the photomicrographs were taken using a Leica DMLB microscope.

### Character Coding and Estimation of Ancestral Character States

To code the characters, we performed comparative anatomical and histochemical analyses of laticifers and phenolic idioblasts for 67 species of Sapindaceae (Supplementary Table 1). The resulting data allowed us to identify variations regarding the presence, anatomical characteristics and cell wall chemical composition, of such secretory structures among species, which were converted into characters and character states. We performed a thorough search for data in the literature on the clades for which we could not obtain biological samples, i.e., *Xanthoceras*, *Doratoxylon*, *Lepisanthes*, *Litchi*, *Nephelium*, *Jagera*, *Guioa*, *Macphersonia*, *Tristiropsis*, and *Haplocoelum*. We retrieved data for *Nephelium*, which was described as having “Phenolic-containing cells” (Landrigan et al., 1994), and *Guioa* (Nielsen, 1991), for which secretory idioblasts were reported. As we detected that phenolic substances are associated with idioblasts in the Sapindaceae as a whole, we coded both genera as possessing phenolic idioblasts. For the other genera no information on laticifers or phenolic idioblasts was found; missing data for these clades were coded using question marks. A list of anatomical and histochemical characters and their state coding used for the ancestral states reconstructions is presented in Table 1.

**TABLE 1:** Phylogenetic characters based on Sapindaceae secretory structures’ characteristics.

Character	Character states
Presence of laticifers	(0) absent, (1) exclusively primary, (2) exclusively secondary, (3) both types
Callose in the laticifer walls	(0) absent, (1) present
Suberin in the laticifer walls	(0) absent, (1) present
Presence of secretory idioblasts	(0) absent, (1) present

Primary laticifers derive from the ground meristem, whereas secondary laticifers originate from the vascular cambium. Despite being derived from different meristems, our analyses showed general anatomical structure and main steps of primary and secondary laticifers development in the family is conserved. We coded the character “Presence of laticifers” discriminating primary from secondary laticifers.

**FIGURE 1:**
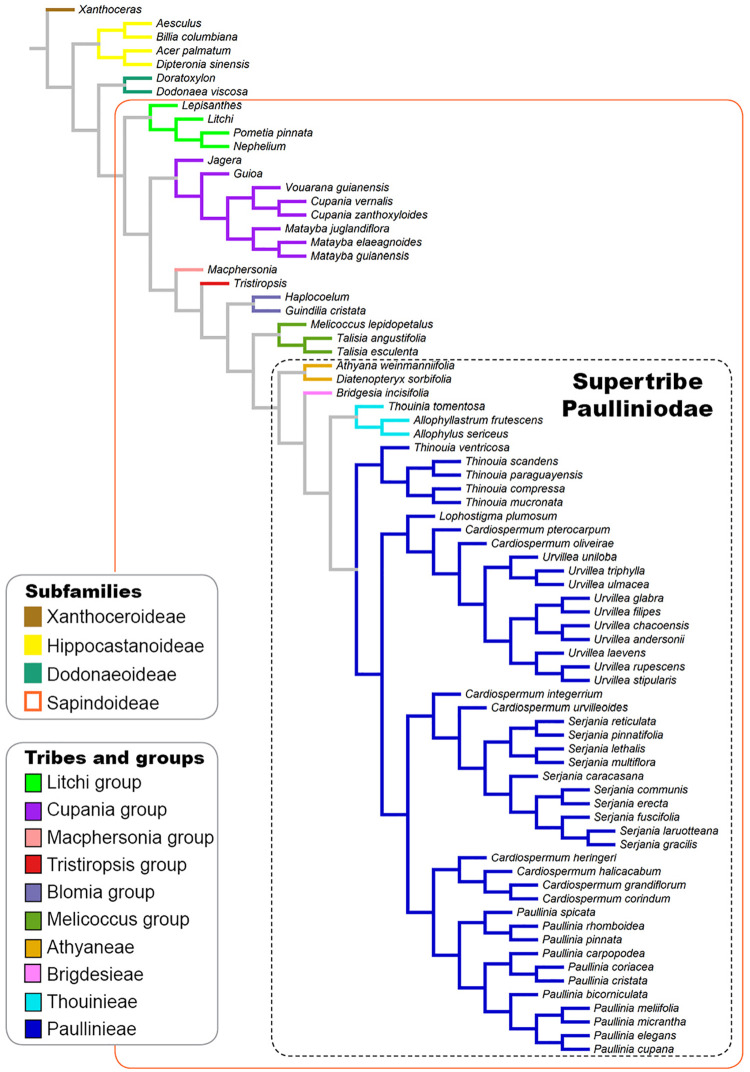
Phylogenetic tree based on the phylogenies published by Buerki et al. (2009) and Acevedo-Rodríguez et al. (2017) showing the infrafamilial and generic relationships in Sapindaceae.

A tree based on the phylogenies published by Buerki et al. (2009); Acevedo-Rodríguez et al. (2017), and Chery et al. (2019) was used to investigate the evolutionary pattern of laticifer characters, as well as the presence/absence of secretory idioblasts in Sapindaceae. A broad and detailed phylogenetic analysis of Sapindaceae is not available. Therefore, we built a synthetic phylogenetic hypothesis, which was constructed in Mesquite (v. 3.6; Maddison and Maddison, 2018). Our informal supertree, comprising 77 terminals, was assembled from the backbone phylogeny in Buerki et al. (2009) and updated, by manually modifying the tree in the software Mesquite, using recently published phylogenetic hypotheses of some individual groups (Acevedo-Rodríguez et al., 2017; Chery et al., 2019; Figure 1). Infrasubfamilial relationships followed the molecular phylogeny of Buerki et al. (2009), which was reconstructed based on plastid and nuclear DNA data. Generic relationships were based on the molecular phylogenies published Acevedo-Rodríguez et al. (2017), which was reconstructed using plastid and nuclear ribosomal markers, and Chery et al. (2019) that was derived from 11 molecular markers, including single-copy nuclear intron markers, plastid *psb*A-*trn*H, and ITS. For this updated version, we added some taxa, removed others, and also changed the position of some, aiming to incorporate more well-resolved relationships from recent studies, as well as to include the taxa for which we had anatomical data. Several Sapindaceae species analyzed in this study have not been formally examined through a phylogenetic approach, thus these taxa were placed on the tree conservatively based on their taxonomic position; we added them to the respective genus/group ancestral node, which resulted in a polytomy that was randomly resolved in the Mesquite software. Character ancestral state reconstructions were estimated from 1,000 iterations of Bayesian stochastic character mapping (Bollback, 2006) using the function make.simmap implemented in the R package phytools (Revell, 2012). A likelihood ratio test (LRT) analysis was performed to each character in order to select the fittest transitional model among the one-parameter, equal-rates model (ER), the symmetric model (SYM), and the all-rates-different model (ARD). We also reconstructed the characters’ states under Maximum Likelihood assumptions using the ace function in the ape package in R (Paradis et al., 2004).

## Results

### Laticifer Distribution

Laticifers were observed in most of the clades of the supertribe Paulliniodae and in *Melicoccus*, *Cupania*, and *Litchi* groups, lineages of the subfamily Sapindoideae sampled in this study. The presence of laticifers was also observed in one genus of Dodonaeoideae and in the four genera of Hippocastanoideae. All laticifers are of articulated non-anastomosing type, and the cell wall thickness is similar to that of the adjacent parenchyma cells or, in some cases, slightly thicker. Depending on the genus, variations were observed concerning the distribution and frequency of laticifers in the plant body, their diameter and the composition of the laticifer wall.

**FIGURE 2:**
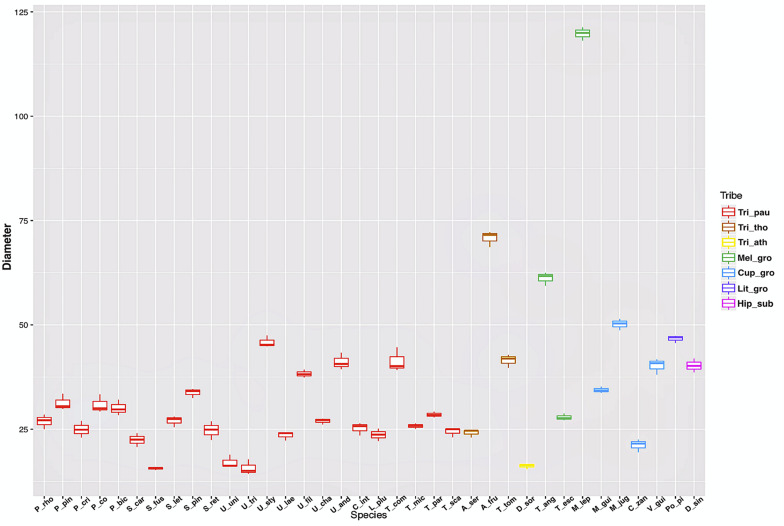
Box plot of laticifer diameters by genus and tribe of Sapindaceae. (Tri_pau, tribe Paullinieae; Tri_tho, tribe Thouinieae; Tri_ath, tribe Athyaneae; Mel_gro, Melicoccus group; Cup_gro, Cupania group; Lit_gro, Litchi Group; Hip_sub, subfamily Hippocastanoideae. P_rho, *Paullinia rhomboidea*; P_pin, *P. pinnata*; P_cri, *P. cristata*; P_co, *P. coriacea*; P_bic, *P. bicorniculata*; S_car, *Serjania caracasana*; S_fus, *S. fuscifolia*; S_let, *S. lethalis*; S_pin, *S. pinnatifolia*; S_ret, *S. reticulata*; U_uni, *Urvillea uniloba*; U_tri, *U. triphylla*; U_sty, *U. stipularis*; U_lae, *U. laevis*; U_fil, *U. filipes*; U_cha, *U. chacoensis*; U_and,U. *andersonii*; C_int, *C. integerrimum*; L_plu, *Lophostigma plumosum*; T_com, *Thinouia compressa*; T_mic, *T. mucronata*; T_par, *T. paraguayensis*; T_sca, *T. scandens*; A_ser, *Allophylus sericeus*; A_fru, *Allophylastrum frutescens*; T_tom, *Thouinia tomentosa*; D_sor, *Diatenopteryx sorbifolia*; T_ang, *Talisia angustifolia*; T_esc, *T. esculenta*; M_lep, *Melicoccus lepidopetalus*; M_gui, *Matayba guianensis*; M_jug, *M. juglandiflora*; C_zan, *Cupania zanthoxyloides*; V_gui, *Vouarana guianensis*; Po_pi, *Pometia pinnata*; D_sin, *Dipteronia sinensis*).

The measurements of laticifer cell diameter showed variations (Figure 2). The two-way ANOVA test revealed that both the tribe (F29, 72 = 1165.4, P less than 2e-16) and the species (F29, 72 = 315.4, P less than 2e-16) to which they belong are statistically significant regarding the diameter of the laticifers, with the tribe being the most significant variable factor, but both influence the diameter of the laticifers analyzed. The means and standard deviations are shown in Figure 2. Species belonging to the tribe Thouinieae (70.81 μm) and the Melicoccus group had the largest recorded diameters, the latter being the one that presented the largest laticifers in the studied species (119.79 μm). The rest of the species remained in a range between 16 and 50 μm. The laticifers of some species of *Urvillea* had the highest diameter average within the tribe Paullinieae (45.85 μm).

#### Subfamily Sapindoideae: Supertribe Paulliniodae

##### Tribe Paullinieae

###### Cardiospermum

**FIGURE 3:**
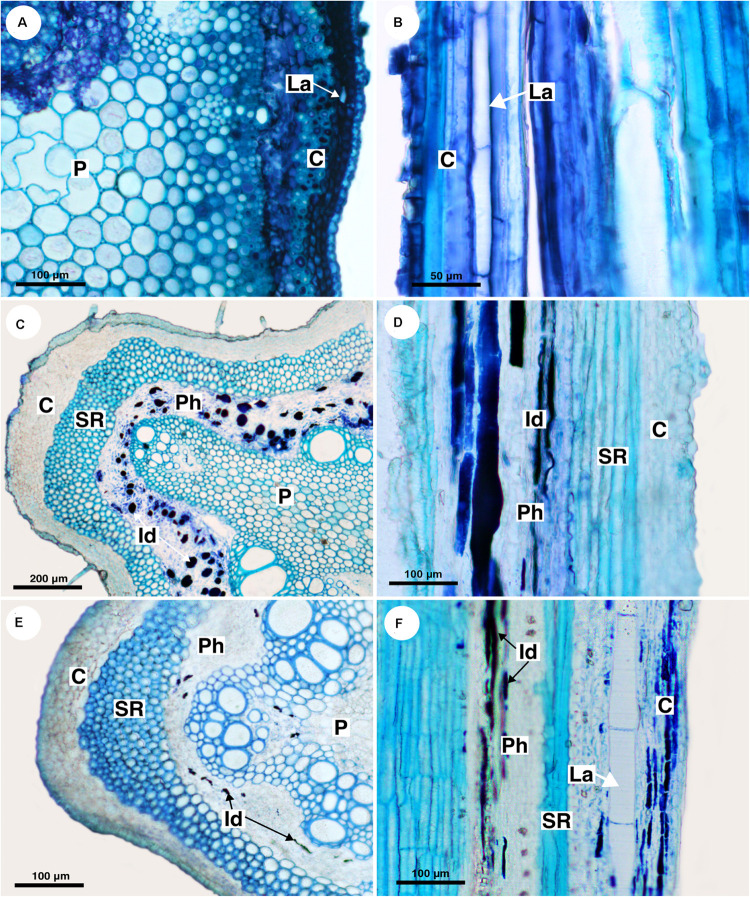
Structure and distribution of laticifers in the supertribe Paulliniodae. Sections stained with toluidine blue. **(A,B)** *Cardiospermum integerrimum.* **(C,D)** *Cardiospermum heringeri.* **(E)** *C. halicacabum.* **(F)** *Lophostigma plumosum.* (C, cortex; Id, idioblast; La, laticifer; P, pith; Ph, phloem; SR, sclerenchyma ring).

**FIGURE 4:**
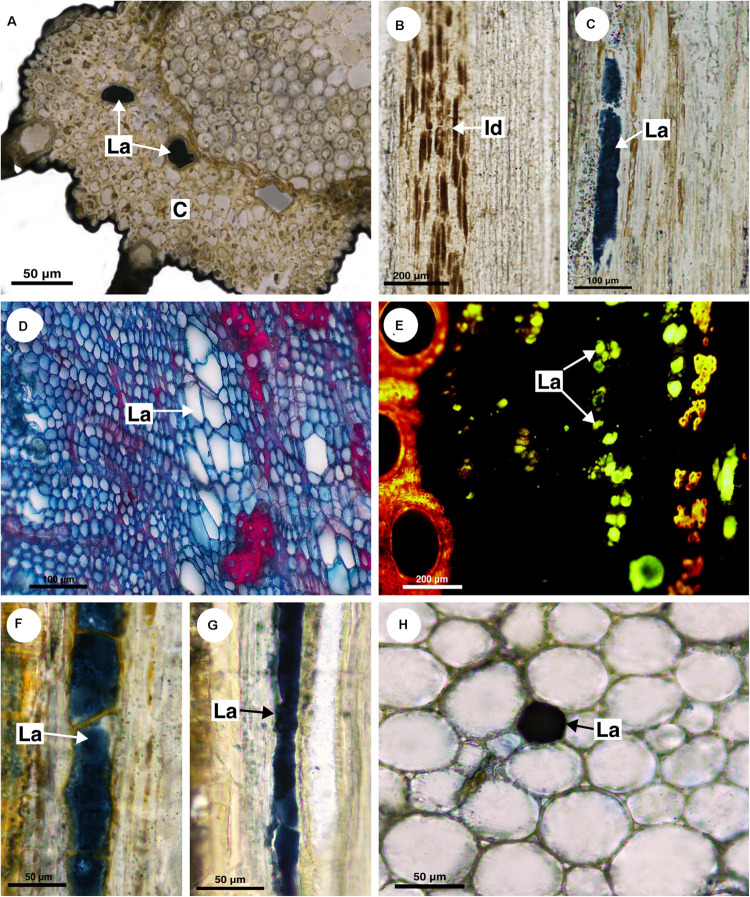
Histochemical analyses of laticifers and idioblasts in Sapindaceae. **(A,C,F–H)** Lipids stained with Sudan black B. **(B)** Phenolic compounds detected by ferric chloride. **(D)** Astra blue and safranin staining. **(E)** Lipids identified by neutral red under blue light. **(A)** *Cardiospermum integerrimum.* **(B)** *C. corindum.* **(C)** *Serjania caracasana.* **(D,E)** *S. laruotteana.* **(F)** *Thinouia paraguayensis.* **(G)** *T. micrantha.* **(H)** *Urvillea chacoensis*. (C, cortex; Id, idioblast; La, laticifer).

Out of eight species of *Cardiospermum* that were analyzed, four have laticifers, i.e., *C. integerrimum*, *C. pterocarpum*, *C. urvilloides*, and *C. oliveirae*. In these species laticifers were observed in the cortex, pith and secondary phloem (Figures 3A,B, 4A).

The other four species (*C. corindum*, *C. grandiflorum*, *C. heringeri*, and *C. halicacabum*) do not have laticifers but presented conspicuous secretory idioblasts, which was confirmed with the ferric chloride test (Figure 4B). They are mainly in the secondary phloem and cortex, where they are near to the sclerenchyma ring (Figures 3C–E). These species have a crystalliferous sheath in the cortex, which loses its crystals during tissue processing for anatomical analysis, becoming very similar to laticifers. Histochemical analyses confirmed that they did not present any type of secretion.

###### Lophostigma

*Lophostigma plumosum* laticifers form long rows in the pith and cortex; they are narrower and longer in the secondary phloem (Figure 3F). Secretory idioblasts were also observed in the phloem.

###### Paullinia

**FIGURE 5:**
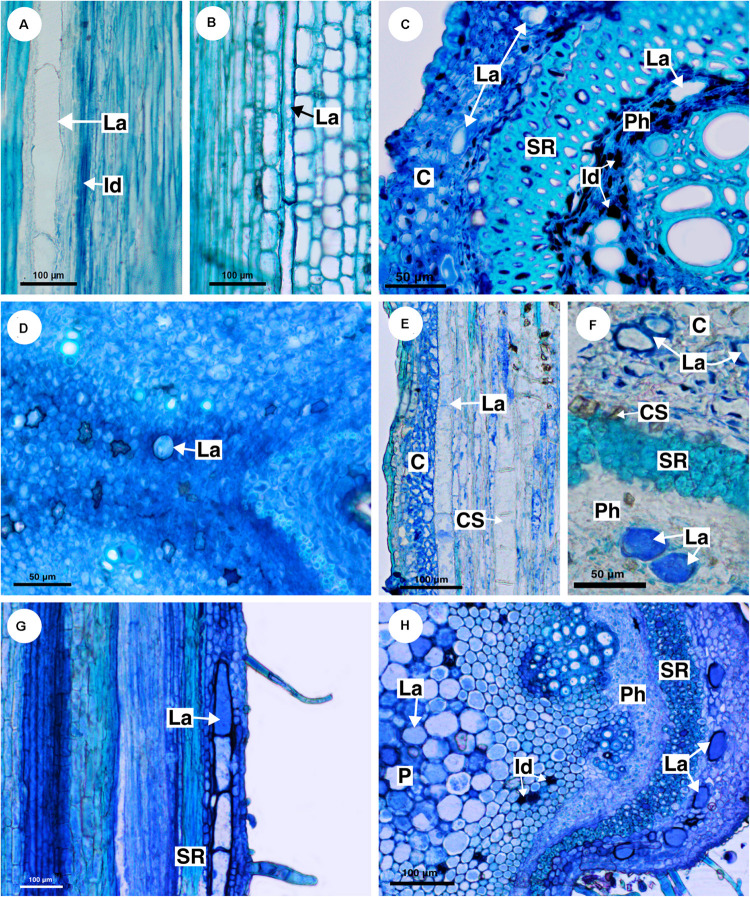
Structure and distribution of laticifers in the supertribe Paulliniodae. Sections stained with toluidine blue. **(A)** *Paullinia bicorniculata.* **(B)** *P. cristata.* **(C)** *Serjania reticulata.* **(D)** *S. fuscifolia.* **(E)** *Thinouia micrantha.* **(F)** *T. paraguayensis*. **(G,H)** *Urvillea filipes.* (C, cortex; CS, crystalliferous sheath; Id, idioblast; La, laticifer; P, pith; Ph, phloem; SR, sclerenchyma ring).

Eleven species of *Paullinia* (*P. corniculata*, *P. carpopoda*, *P. coriacea*, *P. cristata*, *P. cupana*, *P. elegans*, *P. micrantha*, *P. meliifolia*, *P. pinnata*, *P. rhomboidea*, and *P. spicata*) were analyzed and all of them have laticifers. They were observed in the cortex, pith and secondary phloem. When observed in longitudinal section, laticifers in *Paullinia* vary in size. Some are wide and short (Figure 5A), while others are longer and thinner (Figure 5B). When observed in transversal section, the difference in diameter is only slightly variable (Figure 2: See P_rho, P_pin, P_cri, P_co, and P_bic). Walls are reasonably thick and have callose in their constitution (Table 2).

**TABLE 2:** Histochemical tests for detection of compounds present in the latex of Sapindaceae.

Histochemical test	Compound	Laticifers			Idioblasts		
		*P*	*U*	*S*	*P*	*U*	*S*
Sudan black B	Lipids	+	+	+	−	−	−
Sudan IV	Lipids	+	+	+	−	−	−
Neutral red	Lipids	+	+	+	−	−	−
Nile blue	Acidic and neutral lipids	+	+	+	−	−	−
Nadi reagent	Essential oils and resins	+	+	+	−	−	−
Copper acetate and rubeanic acid	Fatty acids	+	+	+	−	−	−
Tannic acid and ferric chloride	Mucilage	−	−	−	−	−	−
Ruthenium red	Acidic mucilage	+	+	+	−	−	−
PAS reaction	Carbohydrates	+	+	+	−	−	−
Aniline blue black	Proteins	+	+	+	−	−	−
Aniline blue	Callose	+	−	+	−	−	−
Wagner’s reagent	Alkaloids	+	−	−	−	−	−
Dragendorff’s reagent	Alkaloids	+	−	−	−	−	−
Vanillin-hydrochloric acid	Tannins	−	−	−	+	+	+
Ferric chloride	Phenolic compounds	+	+	+	+	+	+
Ferrous sulfate in formalin	Phenolic compounds	−	+	+	+	+	+
Potassium dichromate	Phenolic compounds	−	−	−	+	+	+
Lugol’s reagent	Starch	−	−	−	−	−	−

*P, Paullinia seminuda; S, Serjania caracasana; U, Urvillea ulmacea.*

###### Serjania

Ten species of *Serjania* (*S. caracasana*, *S. communis*, *S. erecta*, *S. ruscifolia*, *S. gracilis*, *S. laruotteana*, *S. lethalis*, *S. multiflora*, *S. pinnatifolia*, and *S. reticulata*) were analyzed, and all of them have laticifers, which were observed in the cortex, pith and secondary phloem. In general, laticifers located in the cortical zone and pith are small to medium-sized and scarce. On the other hand, laticifers found in the secondary phloem, where idioblasts are also present, are large (Figures 5C,D). Histochemical tests (Figures 4C–E) confirmed the presence of latex. In this genus cell walls are also thick and have callose (Table 2).

###### Thinouia

Five species of *Thinouia* (*T. compressa*, *T. mucronata*, *T. paraguayensis*, *T. scandens*, and *T. ventricosa*) were analyzed, and all of them have laticifers. In general, the laticifers in this genus are broad and large. In the cortex, they are abundant near the crystalliferous sheath (Figures 5E,F). They are also present in the pith and secondary phloem. The occurrence of latex was histochemically confirmed (Figures 4F,G).

###### Urvillea

Ten species of *Urvillea* (*U. andersonii*, *U. chacoensis*, *U. filipes*, *U. glabra*, *U. laevis*, *U. rufescens*, *U. stipularis*, *U. triphylla*, *U. ulmacea*, and *U. uniloba*) were analyzed, and all of them have laticifers. This genus has laticifers in the cortex, pith, and secondary phloem (Figures 5G,H). In the cortex, they are adjacent to the sclerenchyma ring (Figure 5G). The presence of laticifers was confirmed by histochemical tests (Figure 4H). Secretory idioblasts are also present and are more evident surrounding the pith.

##### Tribe Thouinieae

###### Allophylus

**FIGURE 6:**
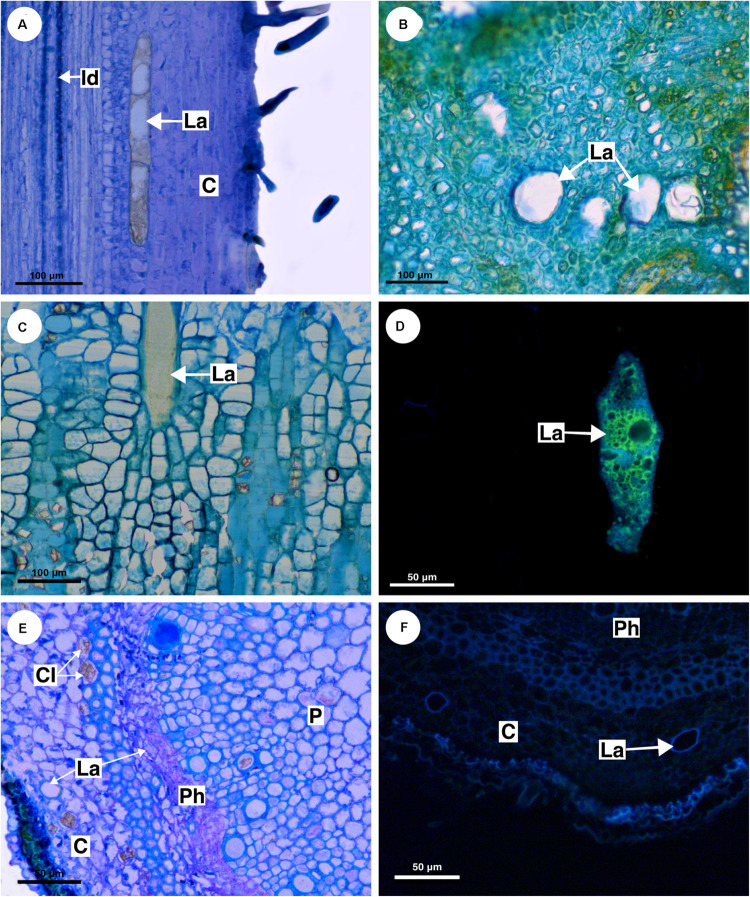
Structure and distribution of laticifers in the supertribe Paulliniodae. **(A)** *Allophylus sericeus*. **(B)** *Allophylastrum frutescens*. **(C,D)** *Thouinia tomentosa*. **(E,F)** *Diatenopteryx sorbifolia.* **(A–C,E)** Toluidine blue staining. **(D,F)** Autofluorescence under UV light. (C, cortex; CI, crystalliferous idioblast; La, laticifer; P, pith; Ph, phloem).

*Allophylus sericeus* laticifers are present in the pith, cortex, and secondary phloem, being larger in the cortex and pith. In longitudinal section, the laticifers are wide and long in comparison to parenchyma cells (Figure 6A). The secretory idioblasts are present in pith and phloem but are more numerous in the secondary phloem.

###### Allophylastrum

Similar to *Allophylus sericeus*, the laticifers in *Allophylastrum frutescens* are located in pith, cortex and secondary phloem (Figure 6B).

###### Thouinia

In *T. tomentosa*, laticifers are wide and short (Figure 6C) forming rows of a few cells. Their walls showed an unusual thickness due to the presence of suberin (Figure 6D). The secretory idioblasts are very small compared with laticifers, and they are present mainly in the secondary phloem.

##### Tribe Bridgesieae

###### Bridgesia

**FIGURE 7:**
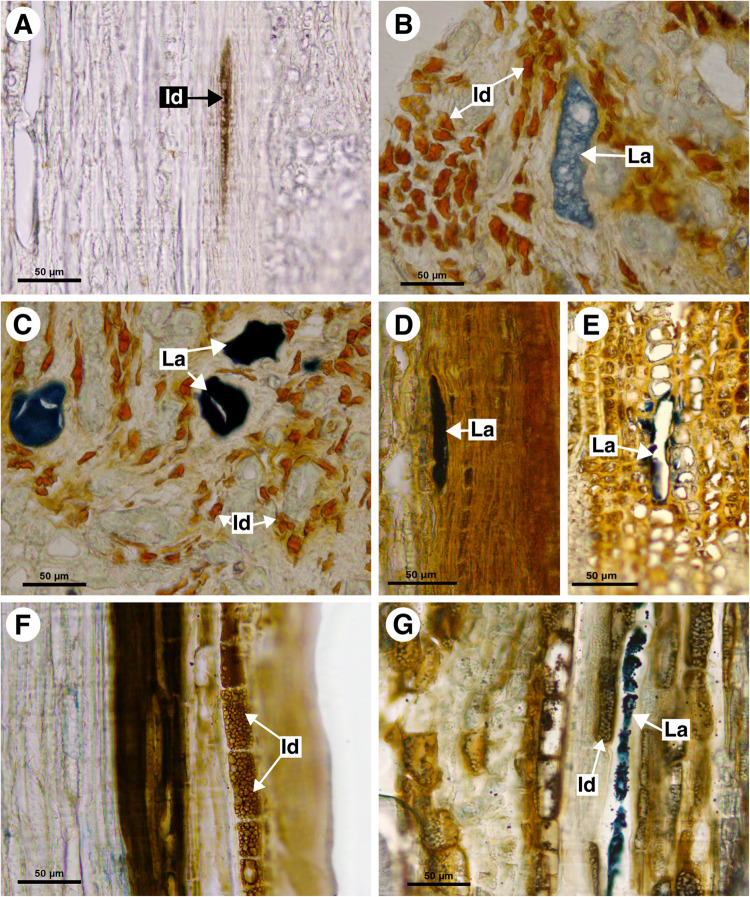
Histochemical analyses of laticifers and idioblasts in Sapindaceae. **(A,F)** Phenolic compounds detected by ferric chloride. **(B–E,G)** Lipids stained with Sudan black B. **(A)** *Bridgesia incisifolia*. **(B,C)** *Matayba elaeagnoides*. **(D,E)** *Cupania zanthoxyloides*. **(F)** *Guindilia cristata*. **(G)** *Dipteronia sinensis.* (Id, Idioblast; La, laticifer).

This monospecific genus does not have laticifers. Few secretory idioblasts were observed in *Bridgesia incisifolia* forming short rows with a maximum of two cells (Figure 7A).

##### Tribe Athyaneae

###### Athyana

This monospecific genus does not have laticifers. However, small secretory idioblasts were identified in *Athyana weinmanniifolia* using ferric chloride.

###### Diatenopteryx

In *D. sorbifolia* laticifers were observed in the cortex and secondary phloem. They were scarce, forming short rows. Cell walls of laticifers, as well as of crystalliferous idioblasts, are thicker compared to parenchyma cells (Figure 6E). In this case, polarized light and Sudan black B test were essential to distinguish laticifers from crystalliferous idioblasts because the latter are large when compared to the parenchyma cells and can be mistaken for laticifers.

Suberin was detected in the laticifer cell wall under UV light (Figure 6F). Secretory idioblasts are abundant in both cortex and pith, forming long rows.

#### Subfamily Sapindoideae: Clades Outside Paulliniodae

##### Melicoccus group

###### Melicoccus

**FIGURE 8:**
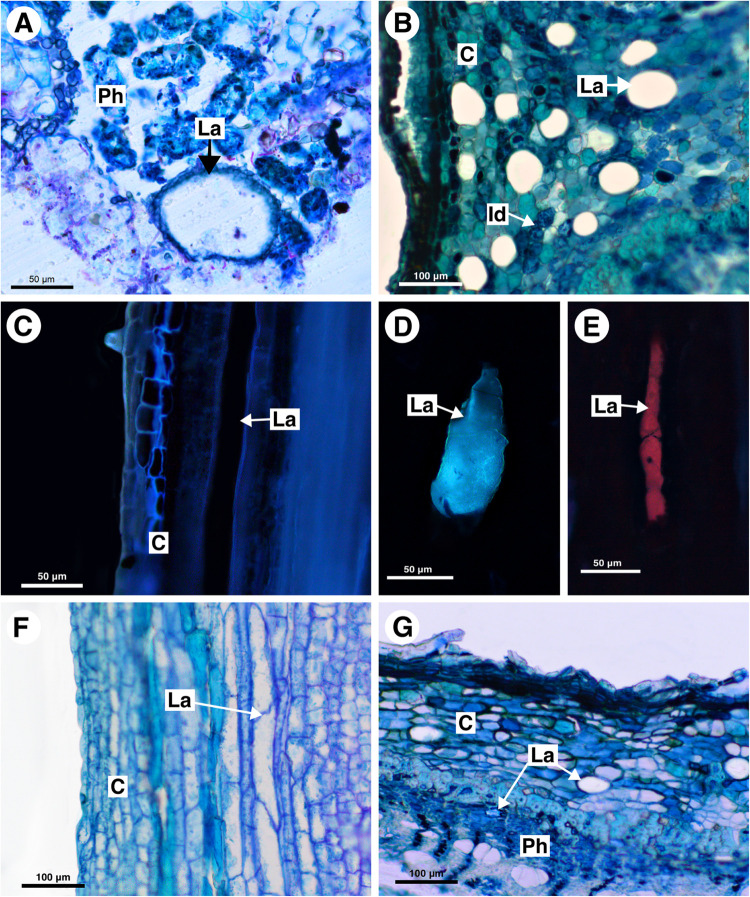
Structure and distribution of laticifers in the Melicoccus group **(A–C)**, Cupania group **(D–F)**, and Litchi group **(G)**. **(A,B,F,G)** Toluidine blue staining. **(C–E)** Autofluorescence under UV **(C,D)** and blue light **(E)**. **(A)** *Melicoccus lepidopetalus*. **(B,C)** *Talisia angustifolia*. **(D)** *Matayba elaeagnoides*. **(E)** *M. juglandiflora*. **(F)** *Vouarana guianensis*. **(G)** *Pometia pinnata*. (C, cortex; Id, Idioblast; La, laticifer; Ph, phloem).

In *Melicoccus lepidopetalus* laticifers were observed in the pith and secondary phloem (Figure 8A).

###### Talisia

In *Talisia angustifolia* and *T. esculenta* laticifers were observed in the cortex, pith and secondary phloem; they are large compared to parenchyma cells (Figure 8B). Suberin was observed in the cell wall (Figure 8C). Idioblasts are distributed in all tissues.

##### Cupania group

###### Matayba

In *M. elaeagnoides*, *M. guianensis*, and *M. juglandifolia* laticifers were observed in both cortex and secondary phloem. They were identified using fluorescence techniques (Figures 8D,E) and the Sudan black B test (Figures 7B,C). The idioblasts are distributed in the cortex, phloem and pith.

###### Vouarana

Laticifers were observed in the cortex, phloem, and pith of *V. guianensis*. Laticifers in the secondary phloem were smaller compared to the ones in the cortex (Figure 8F) and pith. In this genus laticifers are notably more abundant than in other genera of the Cupania group, being conspicuously numerous in the phloem.

###### Cupania

Laticifers in *C. zanthoxyloides* and *C. vernalis* were scarce and were observed forming rows of a few cells (maximum two) in the cortex and pith. Both secondary phloem and cortex laticifers are located near the sclerenchyma ring (Figures 7D,E). Idioblasts were evident in secondary phloem.

##### Litchi group

###### Pometia

In *Pometia pinnata*, laticifers occur in the cortex, pith and secondary phloem; they are long and scarce (Figure 8G). In addition, lipids were also detected using Sudan black B in the ray parenchyma, which likely have a secretory activity.

##### Blomia group

###### Guindilia

*Guindilia cristata* does not have laticifers. It has phenolic idioblasts in the secondary phloem (Figure 7F), which can be confused with laticifers.

#### Subfamily Dodonaeoideae

##### Dodonaea

*Dodonaea viscosa* has laticifers only in cortex located near the sclerenchyma ring. The idioblasts are observed mainly in the phloem and the boundary between the xylem and pith.

#### Subfamily Hippocastanoideae

##### Acer, Dipteronia, Billia, and Aesculus

*Acer palmatum*, *Dipteronia sinensis*, *Billia columbiana*, and *Aesculus hippocastanum* have laticifers exclusively in the secondary phloem, confirmed with the Sudan black B test. Idioblasts were also observed in phloem (Figure 7G).

### Laticifer Development

#### Primary Laticifers

**FIGURE 9:**
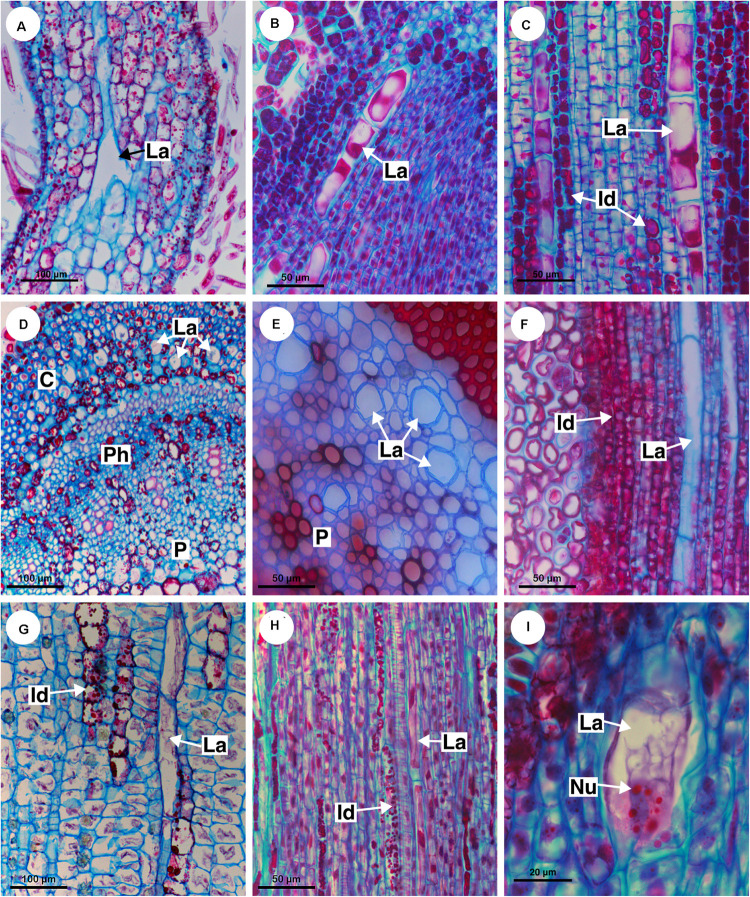
Distribution and ontogeny of laticifers. Sections stained with astra blue and safranin. **(A,F)** *Paullinia seminuda*. Note branched laticifer **(A)**. **(B,C,E,H)** *Serjania caracasana* **(D)** *P. carpopoda*. **(G,I)** *Urvillea ulmacea*. (Id, idioblast; La, laticifer; Nu, nucleoli).

Laticifers and idioblasts were observed in leaves and stems (Figure 9). In stems, both structures are distributed in the cortical and pith areas (Figures 9D,E). Laticifers differ from idioblasts in terms of cell diameter, shape, color, distribution, and aspect of the secretion (Figures 9F,G). The idioblasts are abundant and form extensive rows through the shoot apical meristem, whereas laticifers form less extensive rows and are less abundant when compared with idioblasts (Figure 9F).

All laticifers of Sapindaceae are of articulated non-anastomosing type (Figure 9), i.e., the laticifer is formed by a row of cells which do not fuse, retaining the individuality of each laticiferous cell. Laticifers are unbranched and more or less straight throughout the plant in all species but some branches were found in leaves of *Paullinia* (Figure 9A) and *Serjania*. Both laticifers and idioblasts arise early in the development of the shoot apex, observed among meristematic tissues (Figure 9B). In the studied species, primary laticifers originate from the ground meristem exclusively. Depending on the species, idioblasts can develop before the laticifers or vice versa. Laticifer secretory activity starts right after the first laticiferous cell differentiates, forming a secretion that is clearly visible in the cell lumen (Figures 9B,C).

The first laticifers are mainly located in the cortical region, being formed from the ground meristem near the shoot apical meristem (SAM). They are large when compared with neighboring cells. During the primary growth laticifers accompanying peripherally the vascular system were particularly common (Figure 9H). When compared to idioblasts, laticifers are notably larger (Figures 7C,G,H).

As observed in cross sections, cortical laticifers are formed first, and only when they reach full differentiation are developing laticifers observed in the pith. These are less frequent and composed of cells that are shorter and narrower showing nuclei with numerous nucleoli in *Urvillea* (Figure 9I). In species that form several peripheral vascular cylinders, laticifers are observed in the cortex of each cylinder. In the leaf primordia, laticifers are distributed mainly in the adaxial side.

#### Secondary Laticifers

During secondary growth, the cambium produces laticifers in the phloem (Figures 4D,E, 5A,C, 6E, 8G). The secondary laticifers do not join the laticifers originated during the primary growth. Laticifers originated in the secondary growth form rows that are comprised of fewer cells compared with primary laticifers.

### Chemical Composition of Latex and Laticifer Cell Wall

**FIGURE 10:**
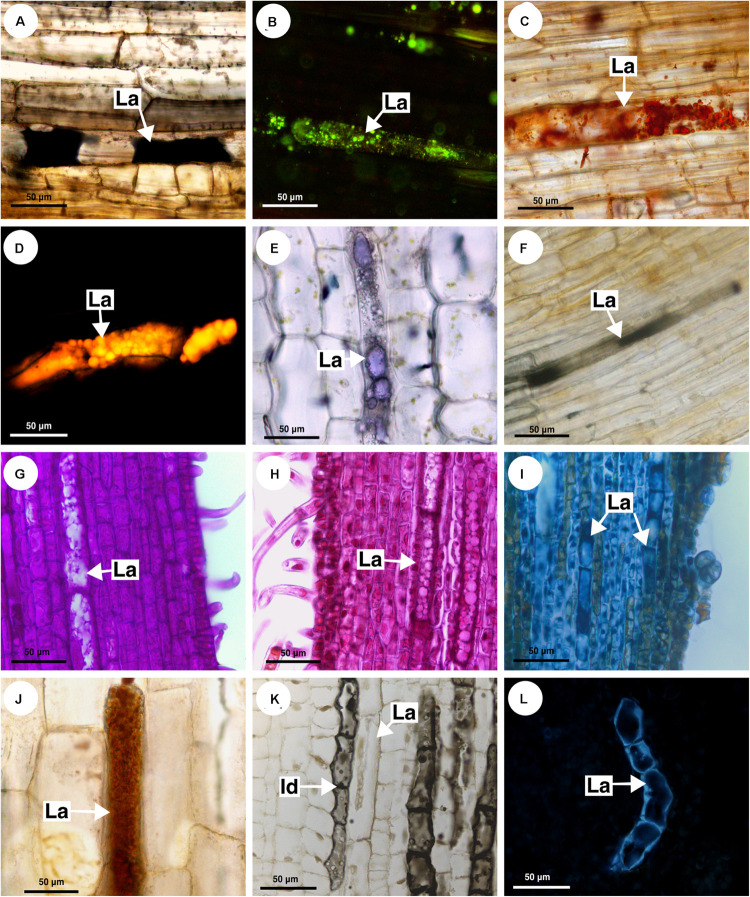
Histochemical analyses of latex and laticifer cell wall in Sapindaceae. **(A,B,F,J)** *Paullinia seminuda.* **(C,I,L)** *Serjania caracasana* **(D,E,G,H,K)** *Urvillea ulmacea.* **(A)** Lipids stained with Sudan black B. **(B)** Lipids detected by neutral red under blue light. **(C)** Lipids identified using Sudan IV. **(D)** Lipids detected by Nile blue under blue light. **(E)** Terpenes identified by Nadi reagent. **(F)** Fatty acids revealed by copper acetate and rubeanic acid test. **(G)** Carbohydrates detected by PAS reaction. **(H)** Acidic mucilage stained with ruthenium red. **(I)** Proteins identified by aniline blue black. **(J)** Alkaloids detected by Dragendorff’s reagent. **(K)** Phenolic compounds revealed by ferrous sulfate in formalin. **(L)** Callose stained with aniline blue under UV. (Id, idioblast; La, laticifer).

The histochemical tests identified a great variety of compounds that constitute the latex. The main component of the latex in Sapindaceae is the lipid fraction (Figures 10A–C), whose predominant compounds were terpenes (Figure 10D), including essential oils and resins (Figure 10E), and fatty acids (Figure 10F). In addition, carbohydrates (Figure 10G), including mucilage (Figure 10H), as well as proteins (Figure 10I) and phenolic compounds, were detected in all species. Alkaloids were identified only in the latex of *Paullinia* (Figure 10J).

Anatomically, the appearance of the exudate from laticifers and idioblasts may be quite similar in some species. Through the ferric chloride test, it became evident that although laticifers may contain phenolic compounds, they are present in proportionally smaller amounts when compared to idioblasts, which specialize in storing phenolic compounds in Sapindaceae (Figure 10K). Phenolic compounds are detected in the latex only by a weak staining in the histochemical tests. Laticifers have primary cell walls that are rich in pectin in all species analyzed. Some genera were distinguished by having callose or suberin in the cell wall (Figure 10L and Table 2).

### Ancestral State Reconstructions

**FIGURE 11:**
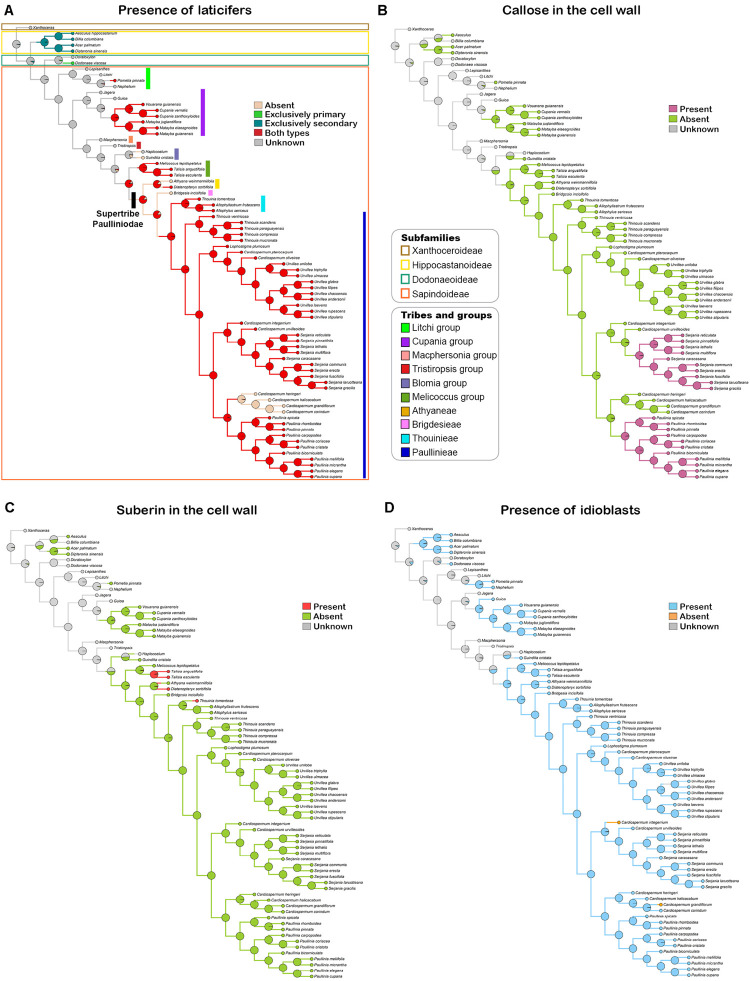
Bayesian stochastic mapping of the character presence of laticifers **(A)**, presence of callose **(B)** and suberin **(C)** in the laticifer cell wall, and presence of secretory idioblasts **(D)**. Pie charts at tree nodes indicate posterior ancestral state probabilities of analyzed characters. Taxonomical information illustrated in figure A apply to all figures.

Based on the anatomical and histochemical analyses, we coded three characters related directly with laticifers and one character derived from secretory idioblasts. A list of morphological characters and their character state coding used for the ancestral state reconstruction is presented in Table 3. Reconstructions of character ancestral states by Bayesian stochastic mapping (Figure 11A) and Maximum likelihood (Supplementary Figure 1A) showed primary, as well as secondary, laticifers had four independent origins in Sapindaceae. The ancestral condition for the family is unknown, whereas secondary laticifers were present in the ancestor of Hippocastanoideae (PP = 0.97; ML = 97%) and primary laticifers evolved in *Dodonaea viscosa* from an ancestor node reconstructed as unknown (PP = 0.95; ML = 95%). Having both types of laticifers is a condition that evolved three times in the family: (1) in *Pometia* from ancestors reconstructed as unknown; (2) in the Cupania group, whose ancestor node was reconstructed as having laticifers (PP = 0.98; ML = 98%); and (3) in the ancestor node of the Paulliniodae + Melicoccus clade that contains the tribe Paullinieae (PP = 0.86; ML = 86%). Three reversals to the ancestral state, i.e., both types of laticifers absent, were observed: (1) in *Athyana*, (2) in *Bridgesia incisifolia*, and (3) in the *Cardiospermum* clade, which is sister group of *Paullinia* (Figure 11A and Supplementary Figure 1A).

**TABLE 3:** Phylogenetic characters derived from laticifers and secretory idioblasts.

Taxon	Laticifers	Callose in the cell wall	Suberin in the cell wall	Secretory idioblasts
*Cardiospermum corindum*	0	0	0	1
*Cardiospermum grandiflorum*	0	0	0	0
*Cardiospermum halicacabum*	0	0	0	1
*Cardiospermum heringeri*	0	0	0	1
*Cardiospermum integerrimum*	3	0	0	0
*Cardiospermum oliveirae*	3	0	0	1
*Cardiospermum pterocarpum*	3	0	0	1
*Cardiospermum urvilleoides*	3	0	0	1
*Lophostigma plumosum*	3	0	0	1
*Paullinia bicorniculata*	3	1	0	1
*Paullinia carpopoda*	3	1	0	1
*Paullinia coriacea*	3	1	0	1
*Paullinia cristata*	3	1	0	1
*Paullinia cupana*	3	1	0	1
*Paullinia elegans*	3	1	0	1
*Paullinia micrantha*	3	1	0	1
*Paullinia meliifolia*	3	1	0	1
*Paullinia pinnata*	3	1	0	1
*Paullinia rhomboidea*	3	1	0	1
*Paullinia spicata*	3	1	0	1
*Serjania caracasana*	3	1	0	1
*Serjania communis*	3	1	0	1
*Serjania erecta*	3	1	0	1
*Serjania fuscifolia*	3	1	0	1
*Serjania gracilis*	3	1	0	1
*Serjania laruotteana*	3	1	0	1
*Serjania lethalis*	3	1	0	1
*Serjania multiflora*	3	1	0	1
*Serjania pinnatifolia*	3	1	0	1
*Serjania reticulata*	3	1	0	1
*Thinouia compressa*	3	0	0	1
*Thinouia mucronata*	3	0	0	1
*Thinouia paraguayensis*	3	0	0	1
*Thinouia scandens*	3	0	0	1
*Thinouia ventricosa*	3	0	0	1
*Urvillea andersonii*	3	0	0	1
*Urvillea chacoensis*	3	0	0	1
*Urvillea filipes*	3	0	0	1
*Urvillea glabra*	3	0	0	1
*Urvillea laevens*	3	0	0	1
*Urvillea rupescens*	3	0	0	1
*Urvillea stipularis*	3	0	0	1
*Urvillea triphylla*	3	0	0	1
*Urvillea ulmacea*	3	0	0	1
*Urvillea uniloba*	3	0	0	1
*Allophylus sericeus*	3	0	0	1
*Allophylastrum frutescens*	3	0	0	1
*Thouinia tomentosa*	3	0	1	1
*Bridgesia incisifolia*	0	0	0	1
*Athyana weinmanniifolia*	0	0	0	1
*Diatenopteryx sorbifolia*	3	0	1	1
*Melicoccus lepidopetalus*	3	0	0	1
*Talisia angustifolia*	3	0	1	1
*Talisia esculenta*	3	0	1	1
*Cupania zanthoxyloides*	3	0	0	1
*Cupania vernalis*	3	0	0	1
*Matayba elaeagnoides*	3	0	0	1
*Matayba guianensis*	3	0	0	1
*Matayba juglandiflora*	3	0	0	1
*Vouarana guianensis*	3	0	0	1
*Pometia pinnata*	3	0	0	1
*Guindilia cristata*	0	0	0	1
*Acer palmatum*	2	0	0	1
*Dipteronia sinensis*	2	0	0	1
*Xanthoceras*	?	?	?	?
*Aesculus hippocastanum*	2	0	0	1
*Billia columbiana*	2	?	?	1
*Doratoxylon*	?	?	?	?
*Dodonaea viscosa*	1	?	?	1
*Lepisanthes*	?	?	?	?
*Litchi*	?	?	?	?
*Nephelium*	?	?	?	1
*Jagera*	?	?	?	?
*Guioa*	?	?	?	1
*Macphersonia*	?	?	?	?
*Tristiropsis*	?	?	?	?
*Haplocoelum*	?	?	?	?

*NA = Not Applicable.*

Callose in the laticifer cell wall evolved twice in the family in *Paullinia* and *Serjania*, both within the tribe Paullinieae. No reversals of this character were observed (Figure 11B and Supplementary Figure 1B). The ancestor of *Paullinia* (PP = 0.98; ML = 98%) and *Serjania* (PP = 0.98; ML = 98%) were reconstructed as possessing callose. Suberin in the laticifer cell wall evolved independently three times in Sapindaceae with no reversals: (1) in *Talisia*, (2) *Diatenopteryx*, and (3) *Thouinia* (Figure 11C and Supplementary Figure 1C).

Secretory idioblasts evolved six times from ancestor nodes reconstructed as unknown in: (1) Hippocastanoideae, (2) *Dodonaea*, (3) an internal node of the Litchi group, (4) an internal clade of the Cupania group, (5) *Guindilia cristata*, and (6) the Paulliniodae + Melicoccus clade (PP = 0.90; ML = 97%). The condition for the family ancestor is unknown, and two reversals to absence of secretory idioblasts from ancestors that had such structure were observed in *Cardiospermum* species (Figure 11D and Supplementary Figure 1D).

## Discussion

This study revealed the presence of laticifers in three subfamilies of Sapindaceae: Hippocastanoideae, Dodonaeoideae and Sapindoideae. Through a detailed anatomical and developmental analysis, we showed that they are of the articulated non-anastomosing type. The anatomical structure of the laticifers in the family is conserved with little or no variation at the species level. However, there are differences among genera, such as the composition of the laticifer cell wall and laticifer size. Using several histochemical tests, we detected that the latex is constituted by compounds from various chemical classes with a prevalence of terpenes. In addition, two laticifer systems occur in the genera analyzed herein: (1) constituted by primary laticifers in the cortex, or in both the cortex and stem pith, and (2) constituted by secondary laticifers present in the secondary phloem. The systems are independent from each other, i.e., they are not interconnected.

### Laticifer Occurrence

Sapindaceae classification has in the past shown some discrepancies in its intergeneric relationships (Acevedo-Rodríguez et al., 2011). More recently, phylogenetic molecular analyses have brought a better understanding of the evolutionary relationships within the family (Kubitzki, 2011; Acevedo-Rodríguez et al., 2017; Chery et al., 2019). Secretory structures exhibit high metabolic complexity, a feature that is conserved among all plant families (Solereder, 1908; Fahn, 1979), and are a potential source of characters for phylogenetic reconstruction. Hence, it is possible that an in-depth anatomical study of secretory structures can lead to a better understanding of the phylogenetic relationships within Sapindaceae at generic and specific levels (Monteiro-Scanavacca et al., 1979; Maleci and Marchi, 1983; Bini Maleci and Servettaz, 1991; Doaigey and Harkiss, 1991; Castro et al., 1997).

Laticifers have been described for only five genera of Sapindaceae: *Paullinia*, *Serjania*, *Urvillea*, *Acer*, and *Dipteronia* (Benedict, 1961; Weckerle and Rutishauser, 2005; Amini et al., 2008; Cunha Neto et al., 2017). In this study, we ascertained the presence of laticifers for the first time in another 12 genera and detected that their presence or absence within genera is constant in Sapindaceae.

Laticifers are lacking in some genera, for instance in *Athyana* and *Bridgesia*. An interesting variation regarding the presence of laticifers was observed in *Cardiospermum*; they are present in *C. integerrimum*, *C. oliveirae*, *C. pterocarpum*, and *C. urvilloides* but absent in *C. corindum*, *C. grandiflorum*, *C. halicacabum*, and *C. heringeri*. These differences support the current circumscription of the genus, in which *Cardiospermum* species that have laticifers were transferred to *Urvillea* or *Serjania* (Acevedo-Rodríguez et al., 2017; see “Ancestral states reconstructions” below).

In the subfamily Hippocastanoideae, specifically in *Acer*, structures called “secretory sacs” in leaf phloem that produce latex have been described (Metcalfe and Chalk, 1950). Taxonomical studies also reported the presence of latex in species of *Acer* (Amini et al., 2008). In *Dipteronia*, a genus with two species, laticifers have also been cited in the phloem of the fruit (Benedict, 1961). In this study, we corroborated the existence of laticifers in the phloem of *Acer* and *Dipteronia*, and describe for the first time the presence of laticifers in *Billia* and *Aesculus*; these four genera of Hippocastanoideae have laticifers located exclusively in the secondary phloem.

For subfamily Dodonaeoideae, the presence of oil channels in the leaf and mucilaginous cells in the epidermis (Manfron et al., 2010) have been described (AL-Aani et al., 2016). However, our work is the first to describe the presence of laticifers in the subfamily, which are found exclusively in the cortex of *Dodonaea viscosa*. Further studies with other genera from Dodonaeoideae are needed to verify whether laticifers are found in other species and, if so, where they are located in the plant body.

Regarding the presence of idioblasts in the family, it has been documented that idioblasts containing saponins, as well as mucilage cells that occur in the leaf epidermis, are common in Sapindaceae (Metcalfe and Chalk, 1950; Kubitzki, 2011; APG, 2016). Apparently, the presence of idioblasts made it difficult to identify laticifers (Milanez, 1959) as they are easily mistaken for one another. Laticifers in Sapindaceae form rows of cells in which the transverse walls do not degrade, making them similar in appearance to rows of idioblasts. It is also difficult to differentiate idioblasts from laticifers in Sapindaceae because in some genera laticifers are composed of short, narrow cells, which is characteristic of idioblasts, whereas in other genera the idioblasts are large and long, being characteristic of laticifers. Nevertheless, as we showed in this study, histochemical analyses clearly separate idioblasts from laticifers as idioblasts always store a substance containing a high percentage of phenolics, while the main chemical compound in latex is lipids. Hence, in some cases, such structures can only be distinguished by the nature of their secretion.

### Latex

In this study we detected that the major component in the latex of Sapindaceae is undoubtedly the lipid portion, as reported for latex of all latescent families (Ramos et al., 2020). It has been reported that the presence or absence of components such as alkaloids may depend on the genus and species. For instance, secretory cells in fruits of *Paullinia cupana* have been classified as secretory tubes with resinous content (Milanez, 1959). However, the content of laticifers of *Paullinia* seems to be much more complex. We detected many different compounds in the latex of *P. carpopoda* and *P. seminuda*, including essential oils, resins, fatty acids, carbohydrates (mainly mucilage), proteins, alkaloids and, in smaller amounts, phenolic compounds. Several studies have demonstrated that latex from *Paullinia* has a wide variety of chemical compounds, among which alkaloids, such as caffeine, theophylline and theobromine, as well as polyphenols, saponins, condensed tannins and cyanogenic compounds, are prevalent (Otobone et al., 2005; Pérez-Dávila et al., 2011). Some species are widely used medicinally in various regions of the Amazon; they are used as pest control in agriculture, as fish poison and as veterinary medication (Iannacone et al., 2007). It is likely that chemotaxonomic studies would find significant differences in the latex composition at the genus and species levels, which could provide relevant taxonomic and phylogenetic characters.

### Ancestral States Reconstructions

#### Presence of Laticifers

Reconstructions of character ancestral states by Bayesian stochastic mapping and Maximum likelihood estimation on the phylogeny of Sapindaceae (Buerki et al., 2009; Acevedo-Rodríguez et al., 2017; Chery et al., 2019) showed laticifers (without discriminating primary from secondary) evolved five times throughout the evolutionary history of the family (Figure 11A and Supplementary Figure 1A). The ancestor of Sapindaceae was reconstructed as unknown, and laticifers emerged multiple times in different lineages of Sapindaceae. For example, the ancestor of the supertribe Paulliniodae + Melicoccus group was estimated to have primary and secondary laticifers. Within this group three reversals occurred. For the supertribe Paulliniodae the probability of the ancestor having both types of laticifers was also high, and the tribes lacking laticifers are small, with few species or monotypic. In addition, the ancestors of the Cupania group and of subfamily Hippocastanoideae were also estimated to have laticifers. In particular, transverse sections showed that laticifer morphology in these groups external to Paulliniodae is peculiar as they are shorter and wider. In the case of Hippocastanoideae, which is a monophyletic group (Buerki, 2010), all genera in the subfamily (*Acer*, *Billia*, *Aesculus*, and *Dipteronia*) have only secondary laticifers, and this character was already present in the ancestor node of the subfamily (97% likelihood).

Altogether, our results revealed that the occurrence of laticifers in Sapindaceae is broader than previously thought. We demonstrate that all supertribe Paulliniodae, as well as species of the Melicoccus group, Cupania group, and Litchi group have laticifers. Latex has never been reported in non-Paulliniodae members of Sapindoideae. We found a few reports of a red exudate in *Pometia* (Litchi group; Kubitzki, 2004), which we detected to be secreted from laticifers after a thorough analysis. Additionally, we also observed ray parenchyma with lipidic content in *Pometia*. A study carried out with *Khaya senegalensis* and *Trichilia cipo* (Meliaceae) showed that the parenchyma ray cells can secrete gum that will eventually be transported to the vessels (De Micco et al., 2016). This process was also observed in 12 species of trees from different families (Saitoh et al., 1993). The composition of gum secreted by ray parenchyma can be complex and is different from the gum produced by secretory ducts (Patten et al., 2010). Usually, gum production has been explained as a response to attacks by pathogens and as a protection from embolism (Saitoh et al., 1993). Secretory idioblasts producing saponins (Suárez et al., 2004) and lipids (Zavaleta-Mancera et al., 2003) have been described in the Cupania group; nonetheless, we also found laticifers in species of this group.

In fact, we identified that laticifers may hold interesting phylogenetic and taxonomic information. For instance, laticifers are secondarily lost in some species of *Cardiospermum* (tribe Paullinieae) but not in others. In the most recent molecular phylogeny for the supertribe Paulliniodae (Acevedo-Rodríguez et al., 2017), *Cardiospermum* emerged as a polyphyletic group: (1) three species formed a monophyletic group that is sister to *Paullinia*, (2) some species formed a monophyletic group with *Serjania*, and (3) some species form a monophyletic group with *Urvillea*. Acevedo-Rodríguez et al. (2017) proposed transferring species from the genus *Cardiospermum* of the item 2 to the genus *Serjania*, and species of the item 3 to *Urvillea*. In our study, we observed that *Cardiospermum s.s*. (*C. corindum*, *C. grandiflorum*, *C. halicacabum*, and *C. heringeri*) does not have laticifers. On the other hand, species of *Cardiospermum* that are closely related to *Serjania* (*C. integerrimum*, *C. urvilleoides*) or that were transferred to *Urvillea* (*C. pterocarpum*, *C. oliveirae*) have laticifers. Hence, our data support their positions as suggested by Acevedo-Rodríguez et al. (2017). Particularly, the close relationship between *Cardiospermum* and *Urvillea* is also supported by the presence of anemochorous septifragal capsules, which are inflated and have a papery pericarp (Weckerle and Rutishauser, 2005).

Possessing laticifers seems to be a widespread condition in Sapindaceae, and gathering data about laticifers for other Sapindoideae groups outside Paullinieae, and for the other three subfamilies (Xanthoceroideae, Dodonaeoideae, and Hippocastanoideae) will contribute not only to a better understanding of their evolution in the family but may also reveal characteristics with phylogenetic and systematic importance.

#### Callose and Suberin in the Laticifer Cell Wall

Callose in the laticifers cell wall evolved twice in the family; both events took place in the tribe Paullinieae and seem to be synapomorphy of *Serjania* and *Paullinia* (Figure 11B and Supplementary Figure 1B). No reversals of this character were observed. Tribe Paullinieae is Neotropical and the most species diverse of the four tribes of Paulliniodae (Acevedo-Rodríguez et al., 2017). The evolution of callose in the laticifer cell wall exclusively in the two most species-rich genera of the tribe Paullinieae (all species of *Paullinia* and *Serjania* species sampled herein had callose), which comprises nearly a quarter of the species in the family, may be interpreted as a potential key innovation that promoted diversification in the tribe. The greater diversification of the tribe Paullinieae has been thought to relate to the evolution of climbing habit, and/or zygomorphic flowers, and/or novel seed dispersal mechanisms (Buerki et al., 2013; Acevedo-Rodríguez et al., 2017).

More effective defense mechanisms could reduce herbivory, allowing for larger population sizes and reduced probability of extinction, thus spurring diversification. Ehrlich and Raven (1964) proposed that new defense strategies result in escape from some herbivores and reduced herbivory; they observed that closely related plants are often attacked by closely related herbivores, a pattern they explained through an “escape and radiate” model, in which after the evolution of a key defense trait, plant lineages diversify, while herbivore lineages diversify along the plant radiation through the development of a key countermeasure (Wheat et al., 2007; Futuyma and Agrawal, 2009). Robust evidence of the “escape and radiate” model has been observed in Brassicaceae, in which shifts in diversification rates in the plant lineages and their insect predators are associated with changes in plant chemical defenses and insect molecular counter adaptations (Edger et al., 2015). In Brassicaceae, defenses evolved from having glucosinolates, which are diagnostic for the family, to possessing additional compounds, such as tropane alkaloids (Brock et al., 2006), cucurbitacins, and cardenolides, in some genera (Futuyma and Agrawal, 2009).

Latex production has been suggested to be a key innovation that has promoted adaptive radiation in plants. The classical study of Farrell et al. (1991) showed that latex-bearing plant taxa were significantly more species rich than their sister clades lacking latex. A recent reassessment of Farrel’s work using an expanded sampling and new information on absence/presence on laticifers and resin ducts found poor support for such structures as general drivers of diversification across plants (Foisy et al., 2019). However, they recognize that this may have occurred in some groups. Using ancestral state reconstructions and phylogenetic models of lineage diversification rates, they detected that acquiring laticifers most likely promoted higher diversification in Papaveraceae. There are also indications that the same phenomenon may have occurred in the clade Euphorbioideae + Crotonoideae, representing two subfamilies of Euphorbiaceae, as well as in Asteraceae, Campanulaceae, Clusiaceae, and Aquifoliaceae. Nevertheless, both studies cited above did not consider the type of laticifer present in each plant group. For instance, plants with articulated non-anastomosing laticifers do not exude latex as profusely as plants with articulated anastomosing laticifers (Demarco and Castro, 2008), which certainly affects the manner in which they interact with predators. Hence, differences in laticifer structure, as well as laticifer particularities, such as presence or absence of callose or suberin in their walls, may have altered diversification rates in distinct ways in the lineages in which they occur.

Suberin is a hydrophobic substance related to the amount of latex released. Laticifer cell walls without suberin in their constitution are permeable to water. Thus, a decrease of turgor pressure in the laticifer due to a plant injury causes an influx of water from adjacent tissues (Downton, 1981), increasing the volume of latex exuded. On the other hand, suberin in laticifer wall prevents water from entering, reducing the volume of latex released and, at times, making it difficult to observe latex in some plants.

Callose is a (1→3)-β-D-glucan synthetized in all kinds of plant tissues in response to wounding (Chen and Kim, 2009). Its accumulation occurs in the cell wall either at injured penetration sites or during infections of fungi, bacteria and viruses (Nakashima et al., 2003; Li et al., 2012; Bellincampi et al., 2014), as well as after feeding by insects (Hao and Wu, 2000; Kuśnierczyk et al., 2008) and nematodes (Hofmann et al., 2010). Deposition of callose is one of the initial mechanisms activated during plant defense against pathogens, and increased accumulation of callose forms a mechanical barrier in the space between the plasma membrane and the cell wall (Heath, 2002; Piršelová and Matušíková, 2013).

It is possible that callose could prevent the entering of viruses and fungi in the laticifer, which may change latex composition and chemical properties profoundly, making it less effective as a defense. For instance, in a study of papaya plants infected with the Papaya meleira virus, the composition of the latex changed; as it became more aqueous, the concentration of proteins and reducing sugars decreased. Latex oxalate raphide-like crystals were remarkably more prominent in infected plants, and it was observed that viral particles were closely associated with such crystals and may use latex to move itself through the plant (Rodrigues et al., 2009). Furthermore, it was shown that that induction of several salicylic acid activated genes, including callose genes, inhibits the development of papaya sticky disease symptoms in papaya plants at the pre-flowering stage (Madroñero et al., 2018).

#### Secretory Idioblasts

Such structures are widespread in Sapindaceae and evolved independently six times from ancestor nodes reconstructed as unknown. The family ancestor was also reconstructed as unknown, and two reversals to absence of secretory idioblasts from ancestors that had such structure were observed in *Cardiospermum* species (Figure 11D). It is possible that the high number of independent origins of this character in the family may be related to the lack of information on groups outside Sapindoideae. Gathering data on secretory idioblasts for these groups may result in an evolutionary pattern with fewer independent origins of such a structure in Sapindaceae.

Our study was the first comprehensive and detailed description of laticifers in Sapindaceae. We show that the occurrence of laticifers in this family is broader than previously reported, with the presence of such secretory structures described for the first time for several genera. The type of laticifer present in the family (articulated non-anastomosing), which does not exude latex profusely when the plant is sectioned, certainly hindered the identification of laticifers in the family. Laticifers had multiple origins in the family and present characteristics that are particular to specific genera, which indicates a metabolic diversity of this structure in the family. Mapping of the character “presence of laticifers” revealed the evolutionary pattern of this character in the family, supporting the new taxonomic circumscriptions recently proposed. Reconstruction of such character showed that groups outside Sapindoideae may have only one type of laticifers, whereas representatives of such subfamily, and in fact the great majority of species in the family, have both types, i.e., primary and secondary laticifers. The evolution of callose in the laticifer cell wall exclusively in the two most species-rich genera of the tribe Paullinieae may be interpreted as a potential key innovation that promoted diversification in the tribe. The greater diversification of Paullinieae has been hypothesized to be the result of the evolution of climbing habit, and/or zygomorphic flowers, and/or novel seed dispersal mechanisms. These features are present in *Paullinia* and *Serjania*; however, they are shared with species-poor clades and thus cannot explain differences in species richness in the tribe. More effective defense mechanisms could reduce herbivory, allowing for larger population sizes and reduced probability of extinction, thus spurring diversification. Further studies on laticifers using a larger sampling, mainly to include the subfamilies Xanthoceroideae, Hippocastanoideae, and Dodonaeoideae, as well as Sapindoideae groups outside Paullinieae, may improve our knowledge of the structure and evolutionary patterns of laticifers, as well as our understanding of the impact of acquiring laticifers on the evolutionary history of the family as a whole.

## Data Availability Statement

The original contributions presented in the study are included in the article/Supplementary Material, further inquiries can be directed to the corresponding authors.

## Author Contributions

MM: data curation, formal analysis, investigation, methodology, writing – original draft, writing, review, and editing. MS-B: data curation, formal analysis, methodology, writing, review, and editing. EP: formal analysis, investigation, and methodology. PA-R: data curation, writing, review, and editing. PD: data curation, writing, review, and editing. DD: conceptualization, data curation, formal analysis, funding acquisition, project administration, supervision, writing – original draft, and writing, review, and editing. All authors contributed to the article and approved the submitted version.

## Conflict of Interest

The authors declare that the research was conducted in the absence of any commercial or financial relationships that could be construed as a potential conflict of interest.
