# Corrigendum: Laticifers in Sapindaceae: Structure, Evolution and Phylogenetic Importance

**DOI:** 10.3389/fpls.2021.669585

**Published:** 2021-03-10

**Authors:** Maria Camila Medina, Mariane S. Sousa-Baena, Erika Prado, Pedro Acevedo-Rodríguez, Pedro Dias, Diego Demarco

**Affiliations:** ^1^Departamento de Botânica, Instituto de Biociências, Universidade de São Paulo, São Paulo, Brazil; ^2^Department of Botany, National Museum of Natural History, Smithsonian Institution, Washington, DC, United States; ^3^Escola de Artes, Ciências e Humanidades – EACH Universidade de São Paulo, São Paulo, Brazil

**Keywords:** laticifers, Paulliniodae, Sapindaceae, evolution, latex, ontogeny, callose, suberin

Publication fee was paid by FAPESP – Fundação de Amparo à Pesquisa do Estado de São Paulo, grant # 2020/16613-5. This information is not in the original article, as the grant was obtained after the article publication.

The authors state that this does not change the scientific conclusions of the article in any way. The original article has been updated.

