# Supplementary material for: Laticifers in Sapindaceae: Structure, Evolution and Phylogenetic Importance
Source: Front Plant Sci. 2021 Jan 18;11:612985. doi: 10.3389/fpls.2020.612985 (PMC7849378; doi:10.3389/fpls.2020.612985)
Supplement: Supplementary file 1 [file Table_1.DOCX]

Supplementary Material

# Supplementary Table 1. Sapindaceae species sampled grouped according to Buerki et al. (2009) and Acevedo-Rodriguez et al. (2017).

| **Subfamily/ Tribe or Group**  **(studied genera/ total genera)** | **Species** | **Collector number** | **Voucher number** | **Collection localities** |
| --- | --- | --- | --- | --- |
| **Sapindoideae/ Paullinieae**  **(6/6)** | *Cardiospermum corindum* L. | Urdampilleta, J. D. 328 | UEC 153247 | Barra, BA, Brazil |
|  |  | Harley, R. M. 16254 | UEC 41658 | Lagoa da Eugênia, BA, Brazil |
|  | *C. grandiflorum* Sw. | Obando, S., et al. 5350 | UEC 171513 | São João Batista do Glória, MG, Brazil |
|  |  | Martins, A. B., et al. 31419 | UEC 67565 | Águas da Prata, SP, Brazil |
|  |  | Firetti, F., et al. f5 | UEC 145213 | Mogi-Guaçu, SP, Brazil |
|  |  | Obando, S., et al. 5350 | UEC 171513 | São João Batista do Glória, MG, Brazil |
|  | *C. halicacabum* L. | Urdampilleta, J. D. 263 | UEC 145127 | Imbaú, PR, Brazil |
|  |  | Urdampilleta, J. D. 288 | UEC 153300 | Saltinho em baixo do Pinar, PR, Brazil |
|  | *C. heringeri* Ferrucci | Urdampilleta, J. D. 363 | UEC 153245 | Caminho a Alto da Bananeira, ES, Brazil |
|  |  | Urdampilleta, J. D., et al. 369 | UEC 171423 | Santa Teresa, ES, Brazil |
|  | *C.* cf. *integerrimum* Radlk. | Kin, A. C., et al. 30014 | UEC 066163 | No data |
|  | *C. oliveirae* Ferrucci | Urdampilleta, J. D. 344 | UEC 153291 | Estrada, Jusiape-Rio de Contas, BA, Brazil |
|  |  | Urdampilleta, J. D. 338 | UEC 153248 | Itaguaçu da Bahia, BA, Brazil |
|  | *C. pterocarpum* Radlk. | Urdampilleta, J. D. 321 | UEC 153275 | Sidrolândia, MS, Brazil |
|  | *C. urvilleoides* (Radlk.) Ferrucci | Urdampilleta, J. D. 425 | SFP 193867 | Itaobim, MG, Brazil |
|  | *Lophostigma plumosum* Radlk. | Ferrucci, M. S., et al. 2681 | UEC 168401 | Narciso Campero, Cochabamba, Bolivia |
|  | *Paullinia bicorniculata* Somner | Melis, J. V. 544 | UEC 168566 | Ubatuba, SP, Brazil |
|  |  | Kim, A. C., et al. 30056 | UEC 66166 | Ubatuba, SP, Brazil |
|  | *P. carpopoda* Cambess. | Obando, S. & Urdampilleta, J. D. 330 | UEC 153203 | Ubatuba, SP, Brazil |
|  |  | Urdampilleta, J. D. 435 | UEC 153198 | Santa Teresa, ES, Brazil |
|  | *P. coriacea* Casar. | Obando, S., et al. 239 | UEC 1399646 | Ubatuba, SP, Brazil |
|  |  | Obando, S., et al. 286 | UEC 139946 | Ubatuba, SP, Brazil |
|  | *P. cristata* Radlk. | Obando, S., et al. 300 | UEC 153209 | Guaraqueçaba, PR, Brazil |
|  |  | Hatschbach, G., et al. 50549 | UEC 45098 | Antonina, PR, Brazil |
|  | *P. cupana* Kunth | Obando, S., et al. 353 | UEC 171416 | Ubatuba, SP, Brazil |
|  |  | Urdampilleta, J. D. 281 | UEC 138838 | Piracicaba, SP, Brazil |
|  | *P. elegans* Cambess. | Solís, M. 81 | UEC 52792 | Brotas, SP, Brazil |
|  |  | Francisco, E. M., et al. 28528 | UEC 157783 | Sertaneja, PR, Brazil |
|  | *P. micrantha* Cambess. | Leitão Filho, H. F., et al. 34483 | UEC 80218 | Ubatuba, SP, Brazil |
|  |  | Leitão Filho, H. F., et al. 34474 | UEC 80225 | Ubatuba, SP, Brazil |
|  | *P. meliifolia* Juss. | Obando, S., et al. 352 | UEC 578 | Piracicaba, SP, Brazil |
|  |  | Urdampilleta, J. D., et al. 452 | UEC 171432 | Parati, RJ, Brazil |
|  | *P. pinnata* L. | Macedo, M. & Assumpção, S. 1768 | UEC 36092 | Est. Porto Cercado, MT, Brazil |
|  |  | Prado, A. L. 3264 | UEC 136476 | Poconé, MG, Brazil |
|  | *P. rhomboidea* Radlk. | Spina, A. P. 270 | UEC 99646 | Campinas, SP, Brazil |
|  |  | Urdampilleta, J. D. & Obando, S. 353 | UEC 153171 | Ubatuba, São Paulo, Brazil |
|  | *P. spicata* Benth. | Silva, G. P. & Pereira, J. B. 4522 | UEC 169169 | Cavalcante, GO, Brazil |
|  |  | Silva, G. P., et al. 4762 | UEC 169172 | Cavalcante, GO, Brazil |
|  | *Serjania caracasana* (Jacq.) Willd. | Obando, S. 295 | UEC 139951 | Ubatuba, SP, Brazil |
|  |  | Carmello-Guerrero, S. M., et al. 212 | UEC 139949 | Botucatu, SP, Brazil |
|  |  | Martins, E. 18401 | UEC 85603 | São Sebastião, SP, Brazil |
|  | *S. communis* Cambess. | Grombone-Guaratini, M. T., et al. 138 | UEC 108259 | Campinas, SP, Brazil |
|  |  | Grombone-Guaratini, M. T., et al. 138 | UEC 108254 | Campinas, SP, Brazil |
|  |  | Grombone-Guaratini, M. T., et al. 136 | UEC 108336 | Campinas, SP, Brazil |
|  | *S. erecta* Radlk. | Raher, J. A. 4003 | UEC 14429 | Brasília, Brazil |
|  |  | Salis, S. M. 144 | UEC 052764 | Brotas, SP, Brazil |
|  | *S. fuscifolia* Radlk. | Passos, F. C., et al. FP83 | UEC 79195 | Gália, SP, Brazil |
|  |  | Scotigma, V. A. 810 | UEC 188506 | Nova Odessa, SP, Brazil |
|  | *S. gracilis* Radlk. | Obando, S., et al. 306 | UEC 153179 | Parque Estadual do Cerrado, PR, Brazil |
|  |  | Urdampilleta, J. D., et al. 301 | UEC 153296 | Ponta Grossa, PR, Brazil |
|  | *S. laruotteana* Cambess. | Tarodo, N., et al. 18597 | UEC 43379 | Campinas, SP, Brazil |
|  |  | Passos, F. C., et al. FP62 | UEC 79170 | Gália, SP, Brazil |
|  | *S. lethalis* A. St.-Hil. | Alencar, M. E. 369 | UEC 119855 | Piripiri, PI, Brazil |
|  |  | Urdampilleta, J. D. 355 | UEC 153222 | São Carlos, SP, Brazil |
|  |  | Obando, S., et al. 348 | UEC 171509 | São João Batista do Glória, MG, Brazil |
|  | *S. multiflora* Cambess. | Spina, A. P. 414 | UEC 99878 | Campinas, SP, Brazil |
|  |  | Leitão Filho, H. F., et al. 1670A | UEC 29757 | Poços de Caldas, MG, Brazil |
|  | *S. pinnatifolia* Radlk. | Urdampilleta, J. D., et al. 408 | UEC 153270 | Rio de Contas, BA, Brazil |
|  |  | Bernacci, L. C., et al. 1825 | UEC 78809 | São Roque de Minas, MG, Brazil |
|  | *S. reticulata* Cambess | Hernandes-Bicudo, L. R., et al. 672 | UEC 44213 | Botucatu, SP, Brazil |
|  |  | Makino, H. 35 | UEC 14499 | Ibiuna, SP, Brazil |
|  | *Thinouia compressa* Radlk. | Harley, R. M. 21701 | UEC 42508 | Correntina, BA, Brazil |
|  |  | Harley, R. M. 21997 | UEC 42518 | Tabovas, BA, Brazil |
|  | *T. mucronata* Radlk. | Leitão Filho, H. F., et al. 23238 | UEC 181267 | Jundiaí, SP, Brazil |
|  |  | Urdampilleta, J. D. 230 | UEC 153193 | Londrina, PR, Brazil |
|  | *T. paraguayensis* (Britton) Radlk. | Ferruci, M. S., et al. 2267 | UEC 165249 | Chiquitos, Santa Cruz, Bolivia |
|  | *T. scandens* Triana & Planch | Vervlvet, R. R., et al. 3024 | UEC 171528 | Governador Lindenberg, ES, Brazil |
|  | *T. ventricosa* Radlk. | Hatschbach, G. 42750 | UEC 50876 | Morretes, PR, Brazil |
|  |  | Magnago, L. F. S., et al. 629 | UEC 171542 | Água Branca, ES, Brazil |
|  | *U. andersonii* Ferruci | Urdampilleta, J. D. & Obando, S. 345 | UEC 153290 | Brumado, BA, Brazil |
|  |  | Hatschbach, M. 56644 | UEC 63502 | Imbituba, SC, Brazil |
|  | *Urvillea chacoensis* Hunz. | Hunziker, At. 22940 | UEC 133045 |  |
|  |  | Ferrucci, M. S., et al. 2701 | UEC 167548 | Santa Cruz, Bolivia |
|  | *U. filipes* Radlk. | Ferrucci, M. S., et al. 2595 | UEC 168381 | Chiquitos, Santa Cruz, Bolivia |
|  | *U. glabra* Cambess. | Torres, R. B., et al. 335 | UEC 151416 | Angatuba, SP, Brazil |
|  |  | Urdampilleta, J. D. & Obando, S. 293 | UEC 153309 | Parati, RJ, Brazil |
|  | *U. laevis* Radlk. | Melis, J. van., et al. 3749 | UEC 168536 | Campinas, SP, Brazil |
|  |  | Ferrucci, M. S., et al. 2534 | UEC 165225 | Chiquitos, Santa Cruz, Bolivia |
|  | *U. rufescens* Cambess. | Wilson Hoenhe 6094 | UEC 14477 | Araruama, RJ, Brazil |
|  |  | Urdampilleta, J. D. & Obando, S. 291 | UEC 153310 | Parati, RJ, Brazil |
|  | *U. stipularis* Ferrucci | Urdampilleta, J. D. 443 | UEC 171424 | Linhares, ES, Brazil |
|  | *U. triphylla* Radlk. | Urdampilleta, J. D. & Obando, S. 349 | UEC 153165 | Ubatuba, SP, Brazil |
|  |  | Urdampilleta, J. D. & Obando, S. 348 | UEC 153166 | Ubatuba, SP, Brazil |
|  | *U. ulmacea* Kunth | Melo, E., et al. 2849 | UEC 131872 | Jaguaquara, BA, Brazil |
|  |  | Obando, S., et al. 310 | UEC 153175 | Jaguariaíva, PR, Brazil |
|  | *U. uniloba* Radlk. | Jarenkow, J. A. 696 | UEC 81495 | Capão do Leão, RS, Brazil |
| **Sapindoideae/ Thouinieae**  **(3/3)** | *Allophylus sericeus* Radlk. | Urdamilleta, J. D. & Ferrucci, M. S. 438 | UEC 171428 | Santa Teresa, ES, Brazil |
|  |  | Martins, F.R. 10056 | UEC 14295 | Santa Rita do Passa Quatro, SP, Brazil |
|  | *Allophylastrum frutescens* Acev.-Rodr. | Perdiz, R. O. 1310 | MIRR 10057 | Boa Vista, Roraima, Brazil |
|  | *Thouinia tomentosa* DC. | Avecedo-Rodriguez, P. 12867 | US | Dominican Republic |
| **Sapindoideae/ Bridgesieae**  **(1/1)** | *Bridgesia incisifolia* Bertero ex Cambess. | Killip, Pisano 39778 | US | Aconcagua, Chile |
| **Sapindoideae/ Athyaneae**  **(2/2)** | *Athyana weinmanniifolia* (Griseb.) Radlk. | Acevedo-Rodriguez, P. 11166 | US 3579590 | Santa Cruz, Bolivia |
|  | *Diatenopteryx sorbifolia* Radlk. | Tamashiro, J. Y., et al. 4718 | UEC 60881 | Campinas, SP, Brazil |
|  |  | Jarenkow, J. A. 945 | UEC 81468 | Marcelino Ramos, RS, Brazil |
| **Sapindoideae/ Melicoccus group**  **(2/2)** | *Melicoccus lepidopetalus* Radlk. | Ferrucci, M. S. 1539 | SPF 149916 | Bella Vista, Amambay, Paraguay |
|  | *Talisia angustifolia* Radlk. | Bernacci, L. C. 20850 | UEC 50176 | Itirapina, SP, Brazil |
|  |  | Semir, J. et al. 11551 | UEC 25443 | São Carlos, SP, Brazil |
|  | *Talisia esculenta* (A. St.-Hil.) Radlk. | Leitão Filho, H. F. 6051 | UEC 14486 | Campinas, SP, Brazil |
| **Sapindoideae/ Cupania group**  **(3/42)** | *Cupania zanthoxyloides* Cambess. | Galvão, J. C. 27111 | UEC 77227 | Atibaia, SP, Brazil |
|  | *C. vernalis* Cambess. | Caselli, C. B. & Schramm, J. E. 42008 | UEC 152339 | Jundiaí, SP, Brazil |
|  | *Matayba elaeagnoides* Radlk. | Tamashiro, J. Y. & Goularti A. M 1320 | UEC 25650 | Poços de Caldas, MG, Brazil |
|  | *M. guianensis* Aubl. | Oliveira, M. M. A. 1993 | UEC 193321 | Aracruz, ES, Brazil |
|  | *M. juglandiflora* Radlk. | Leitão Filho, H. F., el tal. 1162 | UEC 85413 | No data |
|  | *Vouarana guianensis* Aubl. | Nascimento, J. R & Silva, C. F. 624 | UEC 112908 | Itacoatiara, AM, Brazil |
| **Sapindoideae/ Litchi group**  **(1/18)** | *Pometia pinnata* J. R. Forst. & G. Forst. | Kadir A664 | US 3223206 | Kabili, Borneo, Malaysia |
| **Sapindoideae/ Bloomia group**  **(1/2)** | *Guindilia cristata* (Radlk.) Hunz. | Ferrucci, MS. et al. 2930 | US 3628155 | San Juan, Argentina |
| **Dodonaeoideae**  **(1/18)** | *Dodonaea viscosa* Mill. | Wasum, R. s.n. | SPF 152852 | Taqueara, RS, Brazil |
| **Hippocastanoideae**  **(4/5)** | *Acer palmatum* Raf. | Medina, M.C. 0112 | SPF | São Paulo, SP, Brazil |
|  | *Dipteronia sinensis* Oliver | Chun, W.V. 4455 | US | Hupeh, China |
|  | *Billia columbiana* Pevr. | Davidse, G. 21328 | MBM 89790 | Venezuela |
|  | *Aesculus hippocastanum* L. | Smith, A. E. 16A | MBM 97474 | Whiteknights Park, United Kingdom |

Note: MBM, Museu Botânico Municipal; MIRR, Herbário do Museu Integrado de Roraima; UEC, Universidade Estadual de Campinas; SPF, Herbário da Universidade de São Paulo; US, Smithsonian Institution.
