# Supplementary material for: Laticifers in Sapindaceae: Structure, Evolution and Phylogenetic Importance
Source: Front Plant Sci. 2021 Jan 18;11:612985. doi: 10.3389/fpls.2020.612985 (PMC7849378; doi:10.3389/fpls.2020.612985)
Supplement: Supplementary file 2 [file Data_Sheet_1.PDF]

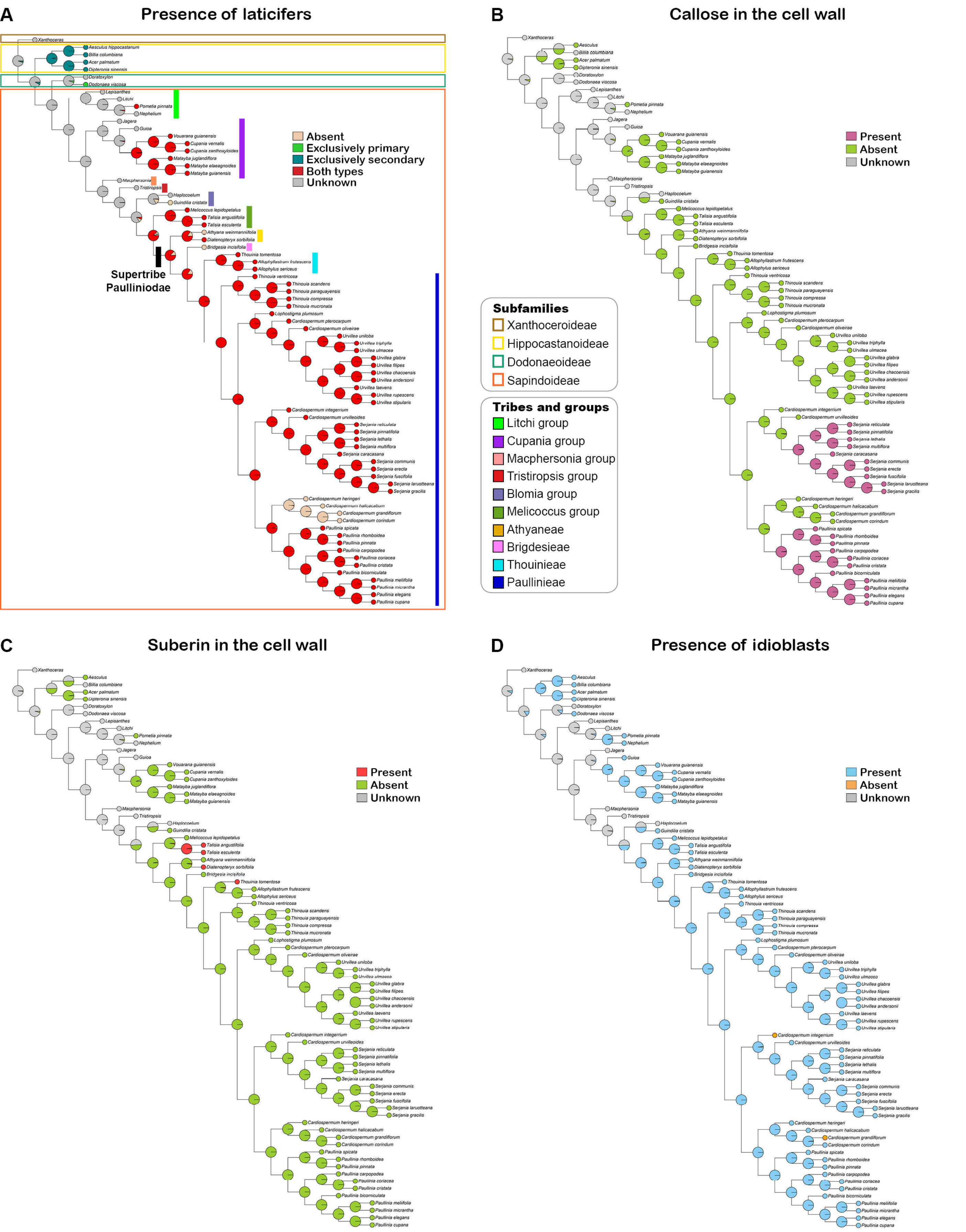

Supplementary Figure 1. Maximum likelihood estimation of the characters presence of laticifers (A), presence of callose (B) and suberin (C) in the laticifer cell wall, and presence of secretory idioblasts (D). Pie charts at tree nodes indicate the likelihoods of analysed characters. Taxonomical information illustrated in figure A apply to all figures.
